# Subinhibitory concentrations of antibiotics affect development and parameters of *Helicobacter pylori* biofilm

**DOI:** 10.3389/fphar.2024.1477317

**Published:** 2024-10-14

**Authors:** Paweł Krzyżek, Paweł Migdał, Kaja Tusiewicz, Marcin Zawadzki, Paweł Szpot

**Affiliations:** ^1^ Department of Microbiology, Faculty of Medicine, Wroclaw Medical University, Wroclaw, Poland; ^2^ Department of Bees Breeding, Institute of Animal Husbandry, Wroclaw University of Environmental and Life Sciences, Wroclaw, Poland; ^3^ Department of Forensic Medicine, Wroclaw Medical University, Wroclaw, Poland; ^4^ Department of Social Sciences and Infectious Diseases, Faculty of Medicine, Wroclaw University of Science and Technology, Wroclaw, Poland

**Keywords:** *Helicobacter pylori*, biofilm formation, biofilm matrix, antibiotic tolerance, antibiotic stress, membrane fatty acids

## Abstract

**Introduction:**

*Helicobacter pylori* causes chronic gastric diseases in nearly 50% of people around the world. It is suggested that biofilm formation has a pronounced effect on the dynamic resistance spread and recurrence of these infections.

**Methods:**

To mimic the scenario of therapeutic ineffectiveness, we investigated the impact of sub-minimal inhibitory concentrations (sub-MICs) of antibiotics on the development and parameters of biofilms produced by clinical *H. pylori* strains.

**Results:**

We observed that constant exposure of planktonic forms to metronidazole or levofloxacin stimulated the speed of autoaggregation and the amount of extracellular matrix, resulting in increased dimensions of the developed biofilms. Contrary to this, continuous exposure to clarithromycin negatively affected a number of biofilm-related reactions and led to the biofilm-weakening effect. Through assessing the membrane fatty acid profiles of antibiotic-exposed cells, we confirmed that metronidazole and levofloxacin induced a biofilm-like phenotype, while clarithromycin kept bacteria in a planktonic form.

**Discussion:**

Our results suggest that sub-MICs of antibiotics affect the biochemical and biophysical properties of the developing biofilm of *H. pylori* strains and may impact the effectiveness of antibiotic treatment.

## 1 Introduction

*Helicobacter pylori* is a Gram-negative, microaerophilic bacterium with a strict adaptation to the human host (Malfertheiner et al., 2023). *H. pylori* colonizes more than half of people around the world and causes chronic gastritis in all of them (Malfertheiner et al., 2022; Tshibangu-Kabamba and Yamaoka, 2021). It is estimated that nearly 90% of gastric cancers and 70%–90% of gastric ulcers are directly related to *H. pylori* infections (Malfertheiner et al., 2022; 2023). Additionally, presence of *H. pylori* within the stomach may be associated with extragastric disorders (neurodegenerative, cardiovascular and metabolic) (He et al., 2022). For this reason, gastritis caused by *H. pylori* is officially recognized by the Maastricht VI consensus as an infectious disease, laying a foundation for the treatment of all patients colonized with this bacterium (Malfertheiner et al., 2022). This postulate changes the long-held paradigm of treatments being reserved only for patients with clinical symptoms. In line with the classification of *H. pylori* gastritis as an infectious disease, the current goal of therapies should be to achieve eradication in the vast majority of infected people (≥95%) (Graham, 2020).

The patient’s response to therapy depends on the antibiotic resistance profile of strains in a given geographical region (Dascălu et al., 2023). The increasing prevalence of resistance to the three most commonly used antibiotics [clarithromycin (CLR), metronidazole (MTZ), and levofloxacin (LEV)] has exceeded the 15% threshold in most regions monitored by the World Health Organization (WHO), leading to the situation when the achievement of therapeutic success is a real challenge (Savoldi et al., 2018; Yu et al., 2024). In light of this, the growing resistance to CLR deserves special attention as CLR-resistant *H. pylori* strains were placed on the WHO list of 12 priority human pathogens (Tacconelli et al., 2018). As extensive meta-analyses and epidemiological studies show, the increasing level of resistance to CLR is associated with a 7-fold increase in the risk of therapeutic failures and is directly correlated with the level of macrolide consumption in the society (Megraud et al., 2021; Savoldi et al., 2018). The most important factors promoting the spread of antibiotic resistance of *H. pylori* include the overuse of antibiotics in the treatment of other infections, poor patient adherence, and factors closely related to the pathophysiology of this bacterium (Dascălu et al., 2023). In this context, the best-known mechanisms of antibiotic resistance in *H. pylori* include chromosomal mutations that disrupt target-mediated activity of drugs (Dascălu et al., 2023; Tshibangu-Kabamba and Yamaoka, 2021). Nevertheless, complex physiological changes promoting tolerance and/or resistance to antibiotics in *H. pylori*, especially biofilm formation, have become increasingly recognized as important (Tshibangu-Kabamba and Yamaoka, 2021).

Biofilm is defined as a conglomerate of microbial cells adhering to each other and immersed in a gel-like, extracellular matrix of various polymeric biomacromolecules (Karygianni et al., 2020; Penesyan et al., 2020). Biofilm is currently perceived as the predominant mode of microbial growth, the importance of which is particularly visible during chronic infections and therapeutic failures (Corona and Martinez, 2013; Penesyan et al., 2020). A growing body of scientific data shows that the effect of antibiotics on the physiology of microorganisms depends on the drug concentration (Penesyan et al., 2020). During repeated exposure of microorganisms to sublethal doses of antibiotics, microbes activate adaptive responses that frequently lead to tolerance toward antimicrobials and, over time, the development of antibiotic resistance (Corona and Martinez, 2013; Sulaiman and Lam, 2021; Windels et al., 2019). The impact of sub-minimal antibiotic concentrations (sub-MICs) on a range of phenotypic traits, including increased abilities to survive treatments, complicates efforts to control infections (Penesyan et al., 2020). Instead of contributing to the destruction of microorganisms, therapeutic agents used incorrectly may enhance microbial survival by promoting the formation of biofilms (Corona and Martinez, 2013; Penesyan et al., 2020). This stimulatory effect of sub-MIC values of antibiotics has noticeable clinical implications, and therefore a better understanding of this phenomenon may improve the effectiveness of therapies aimed at important pathogens infecting humans.

Interest in the topic of biofilm produced by *H. pylori* has undoubtedly flourished in recent years, resulting in the appearance of many research and review works focusing strictly on the description of this phenomenon (Elshenawi et al., 2023; Hathroubi et al., 2018; Hou et al., 2022; Krzyżek et al., 2020; Yonezawa et al., 2015). As described in a detailed review by Tshibangu-Kabamba and Yamaoka (2021), it is indicated that the biofilm formation of *H. pylori* may significantly contribute to the dynamic spread of multidrug resistance, therapeutical ineffectiveness, and the recurrent nature of these infections. Despite this, the current state of knowledge about the development of biofilm *H. pylori* forms is eminently low compared to other clinically important human pathogens (Krzyżek et al., 2020). Recent study performed by our research team (Krzyżek et al., 2022) was focused at deepening the topic of the relationship between biofilm production and antibiotic resistance of clinical *H. pylori* strains. We discovered a significant correlation between biofilm formation and resistance against CLR, but not toward MTZ or LEV. Additionally, we noticed that the strongest biofilm producers had not only a higher tendency for autoaggregation but also a greater production of proteins and eDNA in the biofilm matrix when compared to weak biofilm producers.

Based on the data obtained previously, in the current research article we investigated the impact of sub-MICs of antibiotics on the development and parameters of biofilms in clinical *H. pylori* strains with the most intensive biofilm properties.

## 2 Results and discussion

### 2.1 Selection of *H. pylori* strains and optimization of experimental conditions

In the current set of studies, we decided to use three *H. pylori* strains (1CML, 2CML, and 3CML), which based on a series of experiments determining their phenotypic properties were classified by our team as the most intensive biofilm producers (Krzyżek et al., 2022). These strains present resistance against all three antibiotics most commonly used in therapies – CLR, MTZ and LEV (Savoldi et al., 2018; Yu et al., 2024). For this reason, we believe that they will constitute an excellent research model to verify our hypothesis about a strong connection between adaptive responses toward antibiotic stress and the development of antibiotic resistance, as described in reviews focused on other pathogens (Corona and Martinez, 2013; Penesyan et al., 2020; Sulaiman and Lam, 2021). We assume that *H. pylori* strains able to respond rapidly to antibiotic stress through autoaggregation and production of matrix components may generate mechanisms of antibiotic tolerance (e.g., dynamic biofilm formation). We speculate that this phenomenon may consequently contribute to the development of antibiotic resistance in clinical conditions.

The first stage of research began with the determination of minimal inhibitory concentrations (MICs) and minimal bactericidal concentrations (MBCs) of the tested antibiotics (CLR, MTZ and LEV) in broth cultures. Despite the knowledge of the resistance profile of all three tested *H. pylori* strains, in accordance with the EUCAST recommendations, these resistances were determined using solid media and E-tests (EUCAST, 2024). As MIC values ​​received during broth- and agar-based mode of microbial growth differ (Lallemand et al., 2016), hence we decided that this step was necessary. The MIC and MBC values of the tested *H. pylori* strains in liquid cultures are presented in Supplementary Figure S1.

### 2.2 Antibiotics have different effect on planktonic and biofilm forms of *H. pylori*

**FIGURE 1:**
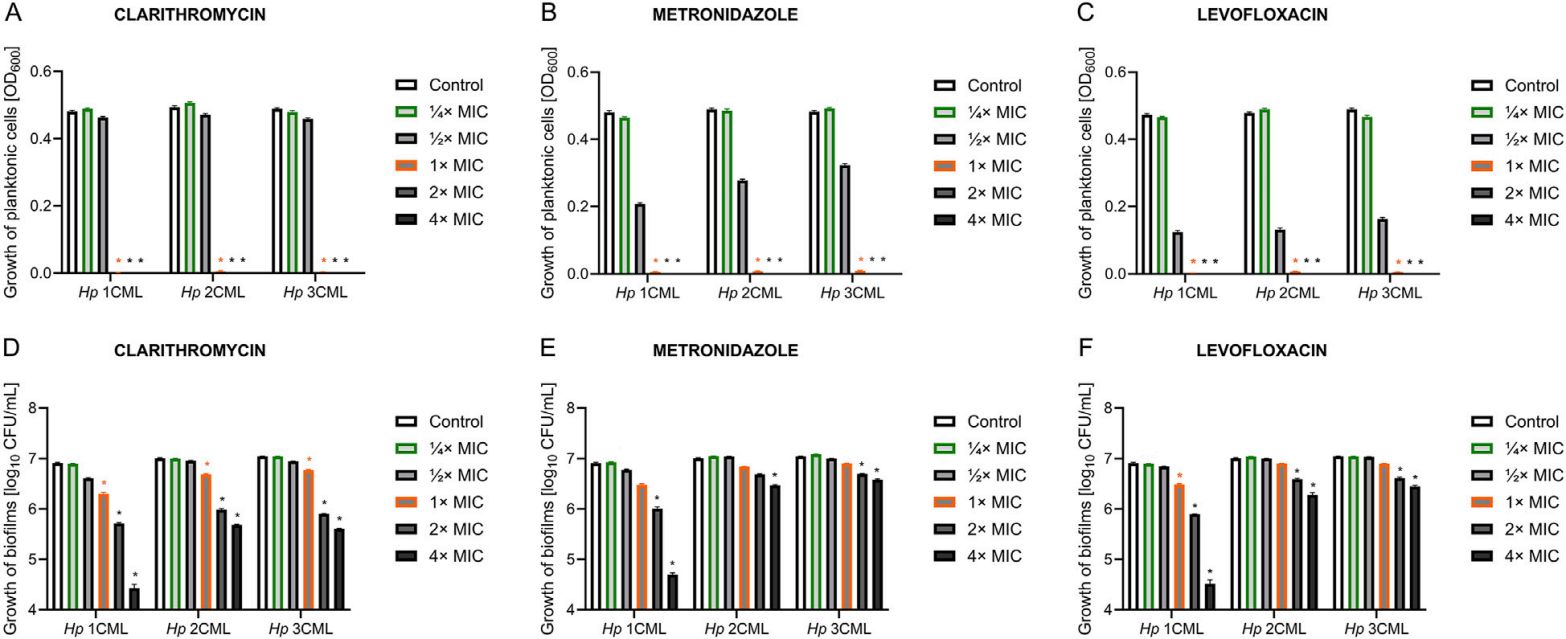
The effect of different antibiotic concentrations on the survival of planktonic and biofilm forms of *H. pylori*. Graphs showing the viability of planktonic forms **(A–C)** and biofilm forms **(D–F)** of the tested *H. pylori* strains exposed to a gradient of antibiotic concentrations (¼× MIC – 4× MIC): clarithromycin **(A**, **D)**, metronidazole **(B**, **E)** and levofloxacin **(C**, **F)**. Bacteria were cultured in 12-well microtiter plates for 3 days at 37°C, microaerophilic conditions and with shaking (100 rpm and 50 rpm for planktonic and biofilm cells, respectively). The viability of planktonic cells was measured using spectrophotometric measurement (OD_600_), and the viability of biofilm cells was measured by sowing biofilm scraped from the walls of microplates and reading CFU/mL after a 3-day culture on Columbia agar at 37°C and microaerophilic conditions. In all cases, values are from three biological replicates with three technical replicates (n = 9); the results are the mean ± standard deviations. Statistical analysis was performed using the Kruskal–Wallis test with Holm correction. p < 0.05 was considered statistically significant.

Having these parameters established, we determined the effect of a concentration gradient (¼× MIC – 4× MIC) of each antibiotic on the viability of planktonic and biofilm forms (Figure 1). As expected, in all three *H. pylori* strains, a 3-day exposure to concentrations in the range between MIC and 4× MIC of each antibiotic contributed to the complete disruption of the growth of planktonic forms (Figures 1A–C). For all antibiotics, both ¼× MIC and ½× MIC had no significant effect on the growth of planktonic forms. Still, however, for MTZ and LEV at ½× MIC some decrease of the viability was seen. For biofilm forms, constant exposure for 3 days had a less significant effect on the reduction in the bacterial viability (Figures 1D–F). For each of the tested antibiotics, the increase in concentration gradually reduced the amount of biofilm, up to a concentration of 4× MIC where a significant disruption in the viability of biofilm was observed. Again, ¼× MIC of each of the tested antibiotics had no significant effect on the biofilm viability.

Based on the results of this pilot study, we selected two concentrations of antibiotics for the next stages of experiments. The ¼× MIC was selected as it did not negatively affect the growth of planktonic and biofilm cells of the tested *H. pylori* strains, constituting an ideal stress-inducing concentration (Corona and Martinez, 2013; Sulaiman and Lam, 2021). The MIC was chosen as the second one as it was able to completely inhibit the growth of planktonic forms of the tested *H. pylori* strains, while had much lower activity against their biofilms. Additionally, this concentration is routinely used in diagnostics as a parameter defining *in vitro* level of susceptibility or resistance to applied antibiotic (EUCAST, 2024), and therefore determining its effect on bacterial physiology is of great clinical importance.

### 2.3 Antibiotics modify the matrix biochemistry of the developing biofilm of *H. pylori*

In the first stage, we verified how a 3-day antibiotic exposure of the tested *H. pylori* strains affects the biochemistry of the biofilm matrix measured through multiple, selective fluorescent staining and microscopic observations. The biofilm matrix is a gel-like polymer surrounding and cementing microorganisms together (Karygianni et al., 2020). The presence of the matrix not only reduces the penetration of drugs into microbial cells, but also enhances chemical interactions between cells and favors the autoaggregation to occur efficiently (Karygianni et al., 2020; Trunk et al., 2018). These types of chemical interactions often take place between polyanionic eDNA molecules and positively charged proteinaceous structures, e.g., secretory proteins, adhesins and flagella (Campoccia et al., 2021; Fong and Yildiz, 2015; Panlilio and Rice, 2021). Recent studies by our team (Krzyżek et al., 2022) and others (Hathroubi et al., 2020b; 2020a; Windham et al., 2018) have shown that the most important components of the biofilm matrix of *H. pylori* are proteins and eDNA. Therefore, we focused on determining whether the amount of these components may be modified when *H. pylori* was exposed to antibiotics.

**FIGURE 2:**
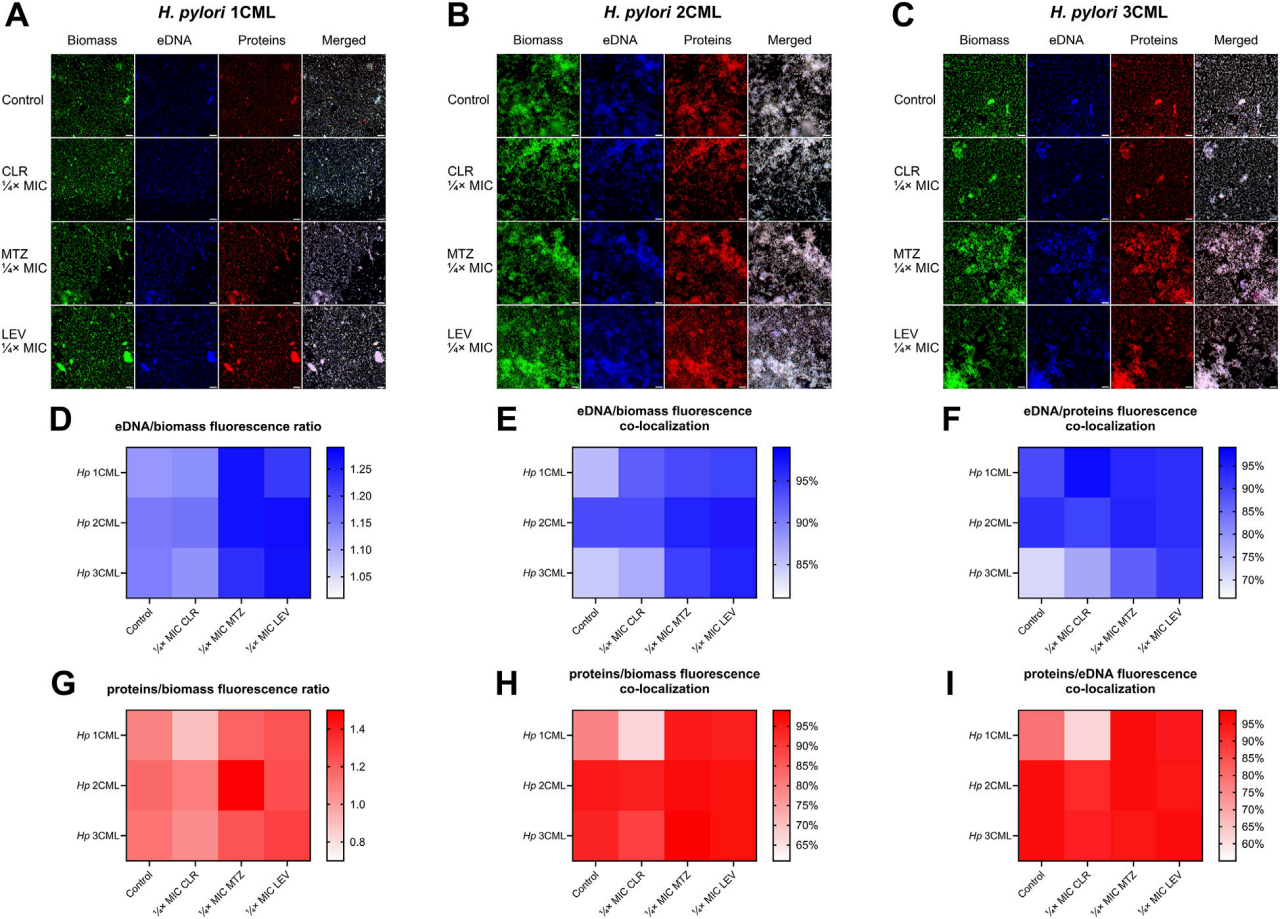
The effect of antibiotics on the biochemical properties of the developing biofilm of *H. pylori*. Schematic panels illustrating representative images of 3-day-old biofilms of *H. pylori* 1CML **(A)**, 2CML **(B)**, and 3CML **(C)** developed under constant exposure to ¼× MIC of antibiotics (clarithromycin [CLR], metronidazole [MTZ] and levofloxacin [LEV]). The development of biofilms took place in 12-well microtiter plates for 3 days at 37°C, microaerophilic conditions and shaking at 50 rpm. Cell biomass, eDNA and proteins were fluorescently stained with SYTO9, DAPI and SYPRO RUBY, respectively. Scale bars = 40 µm. Using the Bioflux Montage software, the fluorescence intensity of biofilm components **(D, G)** and the degree of their co-localization **(E–I)** was calculated and the obtained data were presented as heat maps. Data for eDNA are shown in points **(D-F)**, while data for proteins are illustrated in points **(G–I)**. In all cases, values are from three biological replicates with five technical replicates (n = 15).

We observed that treatment of bacteria with CLR contributed to a reduction in the protein/biomass fluorescence ratio in all *H. pylori* strains, with the effect being stronger at MIC (Figures 2A–F; Supplementary Figure S2A–F). On the other hand, CLR did not significantly affect the eDNA/biomass fluorescence ratio. Exposure of bacteria to ¼× MIC of MTZ or LEV contributed to a significant increase in the fluorescence ratios of both eDNA/biomass and proteins/biomass. The MIC values of these two antibiotics did not change these parameters significantly in most cases.

To obtain more in-depth knowledge about the interactions between individual biofilm components, in the next stage we decided to analyze the degree of their spatial co-localization. This parameter allows for the assessment of the intensity of bacterial coverage by a specific biomatrix component (protein/biomass and eDNA/biomass co-localizations), as well as to estimate the existence of potential interactions between various components of the biomatrix (protein/eDNA and eDNA/protein co-localizations) (Reichhardt and Parsek, 2019).

A detailed analysis of the data allowed us to discover the existence of strain-dependent patterns in the co-localization of biofilm components, while still some of the phenomena were similar between the strains (Figures 2G–I; Supplementary Figure S2G–I). Exposure of *H. pylori* 1CML and 3CML to both concentrations of CLR contributed to the reduction of protein/biomass and protein/eDNA co-localizations. Treatment of these strains with ¼× MIC of MTZ or LEV enhanced co-localizations of eDNA/biomass and proteins/biomass. Some discrepancies were observed when these strains were exposed to MIC concentrations, indicating a strain-dependent type of responses during the presence of high concentrations of antibiotics. A strain with a slightly different behavioral profile was *H. pylori* 2CML, which in our previous study was categorized as the strongest biofilm producer (Krzyżek et al., 2022). In this case, in the control setting, the degree of co-localization of all components was very high (>90%) and, with few exceptions, was not significantly modified after antibiotic treatment (Figures 2G–I; Supplementary Figure S2G–I).

To sum up, we observed that exposure of *H. pylori* to CLR decreased the amount of proteins in the biofilm, whereas treatment with MTZ or LEV intensified the amount of both proteins and eDNA in the biofilm. Moreover, presence of antibiotics was also accompanied by changes in co-localization of the biofilm components, with a weakening or neutral effect for CLR and a stimulatory impact of MTZ and LEV.

We assumed that differences in the molecular mechanism of action of these antibiotics were responsible for these phenomena. CLR, as a representative of macrolides, contributes to the inhibition of translation (Vázquez-Laslop and Mankin, 2018), and therefore may ultimately reduce the amount of proteins in the biofilm matrix of the tested *H. pylori* strains. We confirmed these results independently using a Rapid Gold Pierce BCA protein assay and a spectrophotometric quantification of the protein amount (Supplementary Figure S3). On the other hand, MTZ (nitroimidazoles) and LEV (fluoroquinolones), in addition to their well-known activity targeting DNA, are recognized as strong activators of oxidative stress (Drlica and Zhao, 2021; Thomas and Gwenin, 2021). We confirmed the ability of both antibiotics to induce oxidative stress in the tested *H. pylori* strains using a H2DCFDA staining and fluorescence estimation (Supplementary Figure S4). Based on the above observations, we believe that the ability of MTZ or LEV to induce oxidative stress was responsible for the intensified formation of the biofilm matrix by the tested *H. pylori* strains. Our analysis in this area is well complemented by the data of Zhao et al. (2021), who recently proved the capacity of a widely-known oxidant H_2_O_2_ to cause the oxidative stress-dependent promotion of biofilm development in *H. pylori*.

### 2.4 Antibiotics modify the autoaggregation rate and biophysical parameters of the developing biofilm of *H. pylori*

Although our studies showed many correlations between exposure to antibiotics and production of biofilm matrix components in *H. pylori*, this data did not allow for a sufficient explanation of the biofilm-modulating effect of sub-MICs of antibiotics on the tested strains. Therefore, in the second stage of the research, we undertook to determine the impact of antibiotics on the primary stages of biofilm development – formation of cell aggregates (referred to as autoaggregation). Many microorganisms, both environmental and pathogenic, have the ability to self-aggregate (Trunk et al., 2018). During this process, microorganisms of one species adhere to each other and form multicellular clusters (Nwoko and Okeke, 2021; Trunk et al., 2018). These structures protect against external stressors and antimicrobial substances, and are often considered as a crucial step preceding biofilm formation (Nwoko and Okeke, 2021; Trunk et al., 2018). With this in mind, we performed a time-lapse live analysis of autoaggregation of the tested *H. pylori* strains during their 1-h exposure to antibiotics.

**FIGURE 3:**
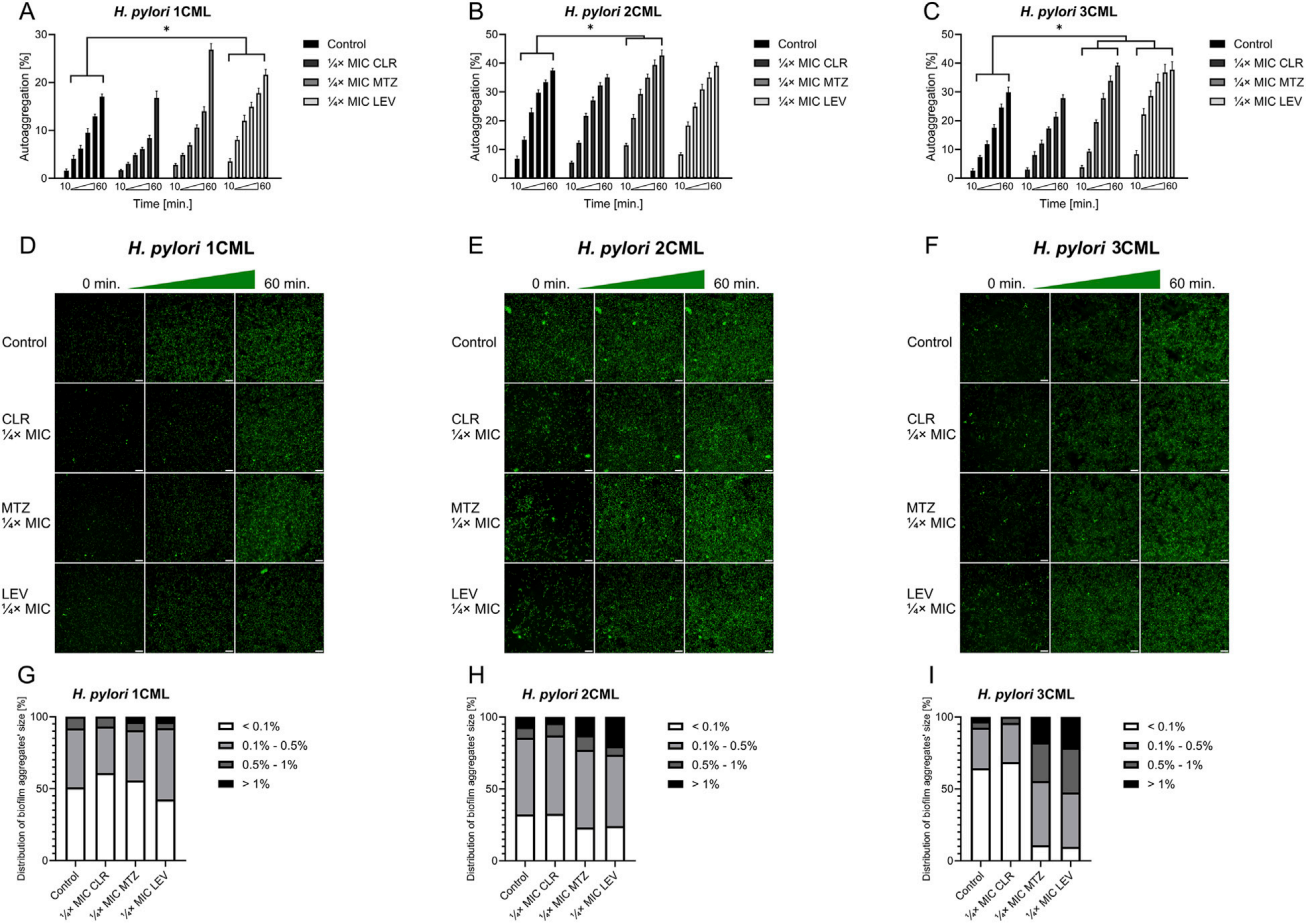
The effect of antibiotics on the autoaggregation of *H. pylori*. Graphs and panels of representative photos showing the speed of autoaggregation of *H. pylori* 1CML **(A, D)**, 2CML **(B, E)** and 3CML **(C, F)** during 1-h exposure to ¼× MIC of antibiotics (clarithromycin [CLR], metronidazole [MTZ] and levofloxacin [LEV]). The autoaggregation experiments took place in 12-well microtiter plates at 37°C and microaerophilic conditions. The speed of autoaggregation was interpreted as the rate of bacterial coverage of the observation field and was calculated using the ImageJ software. The speed of autoaggregation was each time normalized in relation to the initial amount of autoaggregation (0 min), hence the starting point was not included in graphs **(A–C)** and was considered as 0%. For better visualization of the cells, the bacteria were treated with 1 μL/mL of the non-toxic fluorescent dye from the CellTrace CFSE Cell Proliferation Kit. Scale bars for photographic panels **(D–F)** are equal to 40 µm. Using the ImageJ software, additional information was obtained about the size distribution of aggregates, which was presented in **(G–I)** charts. In all cases, values are from three biological replicates with three technical replicates (n = 9); the results are the mean ± standard deviations. Statistical analysis was performed using the Kruskal–Wallis test with Holm correction. p < 0.05 was considered statistically significant.

In the first step, we analyzed the speed of autoaggregation, understood as the number of aggregates covering the observation field at a specific time point (Krzyżek et al., 2022). We noticed an intensification of the speed of autoaggregation when using ¼× MIC of MTZ (*H. pylori* 2CML and 3CML) or ¼× MIC of LEV (*H. pylori* 1CML and 3CML), while treatment with ¼× MIC of CLR had a neutral effect on this process (Figures 3A–F). The MIC values of all tested antibiotics didn’t significantly affect the course of autoaggregation (Supplementary Figure S5A–F). Representative videos showing the autoaggregation of *H. pylori* 2CML in a control setting and samples exposed to ¼× MIC of antibiotics are shown in Supplementary Videos S1–4.

In the next step, we analyzed how a 1-h antibiotic exposure of *H. pylori* strains affects the size distribution of aggregates formed by these bacteria (Figures 3G–I; Supplementary Figure S5G–I). For all three strains, we noticed a shift in the aggregates’ size distribution towards small and medium-sized after treatment with ¼× MIC or MIC of CLR. The opposite situation, consisting in an increase in the number or *de novo* appearance of big and large aggregates, was observed after exposure of all three strains to ¼× MIC of MTZ or LEV. The MIC values of both these antibiotics did not have a stimulatory effect on autoaggregation.

**FIGURE 4:**
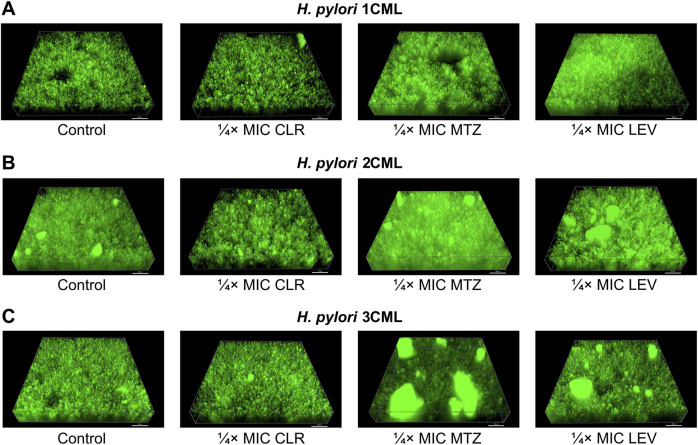
The effect of antibiotics on the biophysical properties of the developing biofilm of *H. pylori*. Panels of representative photos showing biofilms of *H. pylori* 1CML **(A)**, 2CML **(B)** and 3CML **(C)** created during autoaggregation experiments and 1-h exposure of bacteria to ¼× MIC of antibiotics (clarithromycin [CLR], metronidazole [MTZ] and levofloxacin [LEV]). The development of biofilms took place in 12-well microtiter plates at 37°C and microaerophilic conditions. Using the Bioflux Montage software, a total of 50 Z-stacks were collected in each case from the center of the examined well of the microtiter plate. The photographs obtained in this way were processed using the ImarisViewer. Scale bars = 50 µm.

In the further step, we assessed how the tested antibiotic concentrations affect the 3D structure of the biofilm produced after a 1-h exposure (Figure 4; Supplementary Figure S6). This set of analyzes confirmed our previous observations. We noticed that exposure to ¼× MIC or MIC of CLR was accompanied by a reduction in the biovolume of biofilms. When treating bacteria with ¼× MIC of MTZ or LEV, an inverse relationship associated with the intensification of the biofilm biovolume was observed. Once again MICs of both these antibiotics didn’t impact significantly the 3D structure of biofilms.

**FIGURE 5:**
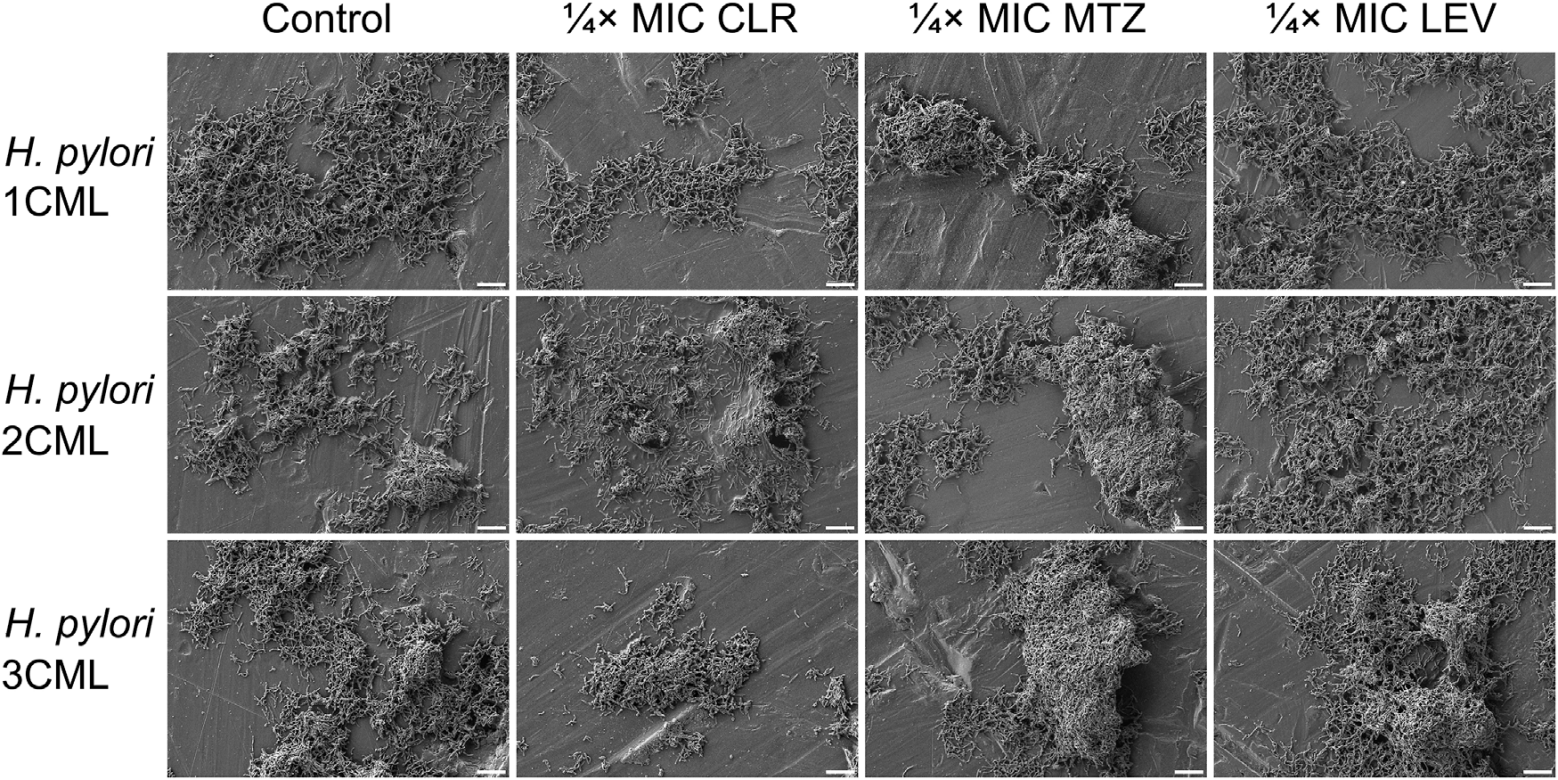
The effect of antibiotics on the ultrastructure of the developing biofilm of *H. pylori*. Panel of representative photos showing biofilms of *H. pylori* 1CML, 2CML and 3CML created during autoaggregation experiments and 1-h exposure of bacteria to ¼× MIC of antibiotics (clarithromycin [CLR], metronidazole [MTZ] and levofloxacin [LEV]). The development of biofilms took place in 12-well microtiter plates at 37°C and microaerophilic conditions. Scale bars = 10 µm.

In addition to the above, we confirmed independently the results covering the impact of 1-h exposure to ¼× MIC of antibiotics on biophysical properties of biofilms using scanning electron microscopy observations (Figure 5). The photographic panel presents modifications of aggregates produced by all three *H. pylori* strains after antibiotic treatment. These changes were seen as small, dispersed aggregates when bacteria were exposed to ¼× MIC of CLR, and highly compact, spatially complex clusters after treatment with ¼× MIC of MTZ or LEV.

To sum up, we noticed that ¼× MIC of MTZ and LEV not only stimulated the speed of *H. pylori* autoaggregation, but also led to the development of biofilms with increased, more spatially complex structure. In the case of exposure to CLR, although there was no effect on the speed of autoaggregation, a negative impact on the aggregates’ dimensions and the biovolume of the biofilm itself was still observed. For MICs of all antibiotics, the aggregation-modulating effect was often lost. Given what have been discovered, in our opinion, the ¼× MIC values of antibiotics are of particular interest as they did not have a negative impact on the multiplication of planktonic and biofilm forms of *H. pylori* (Figure 1), while affected a number of phenotypic features related to the biofilm development (Figures 2–4). Our results on *H. pylori* strains are very similar to the observations of other research teams conducted on various bacterial species. These experiments showed the biofilm-promoting effect of sub-MICs of MTZ against *Clostridioides difficile* (Doan et al., 2022; Vuotto et al., 2016; Wultańska et al., 2023) and sub-MICs of fluoroquinolones against *Pseudomonas* spp. (Liu et al., 2021; She et al., 2019), *Streptococcus pyogenes* (Balaji et al., 2013) or uropathogenic *Escherichia coli* (Rafaque et al., 2020). At the same time, it has been shown that exposure to sub-MICs of macrolides contributes to the reduction in the biofilm amount in many Gram-negative fermentative (Moshynets et al., 2022; Zafar et al., 2020) and non-fermentative (Wozniak and Keyser, 2004; Yamabe et al., 2020) rods.

### 2.5 Microfluidic culture conditions highlight the vital role of autoaggregation in responses of *H. pylori* against antibiotic stress

**FIGURE 6:**
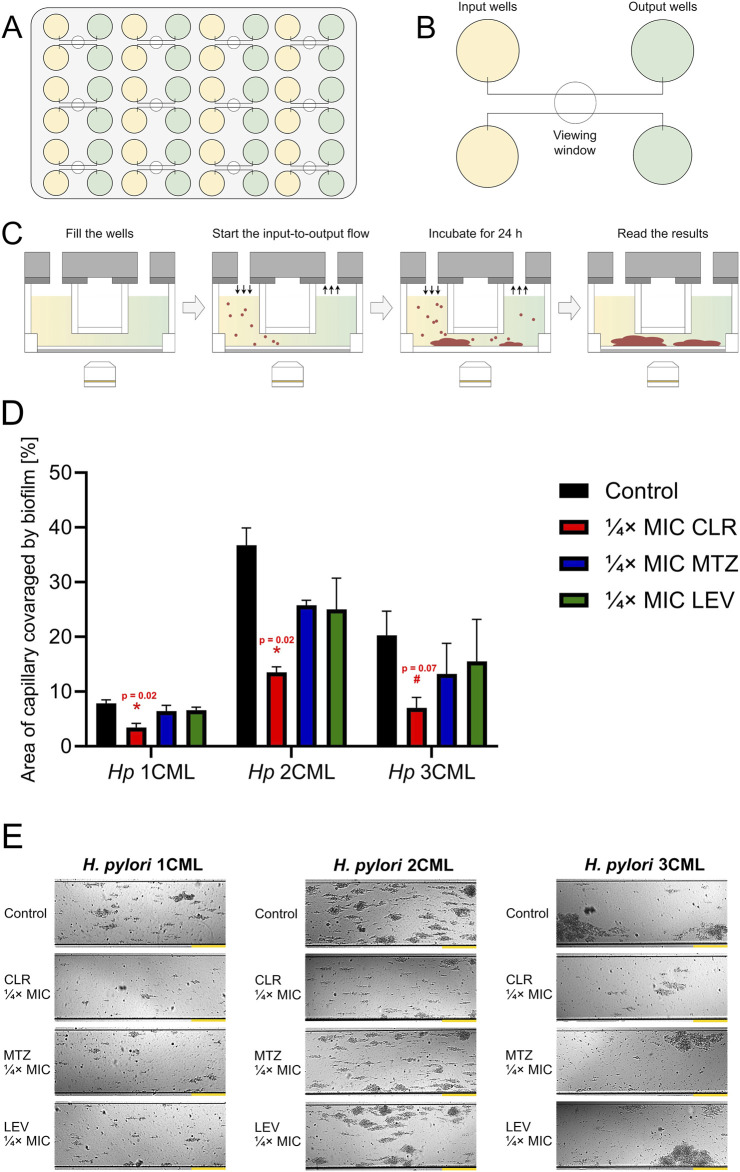
The effect of antibiotics on the biofilm development of *H. pylori* in microfluidic conditions. Cartoon images showing the microfluidic plate **(A)** and a close-up of its fragment containing the inlet and outlet wells connected by microcapillaries **(B)**. Point **(C)** presents the scheme of the experiment, including microfluidic culture of *H. pylori* strains. In point **(D, E)**, a graph showing the degree of development of biofilms of *H. pylori* strains and a panel of representative photos showing the growth of *H. pylori* biofilms in microcapillaries are presented, respectively. Bacteria were cultured in 48-well microfluidic plates for 1 day at 37°C, microaerophilic conditions and medium flow of 0.1 dyne/cm^2^ and were exposed to ¼× MIC of antibiotics (clarithromycin [CLR], metronidazole [MTZ] and levofloxacin [LEV]). The degree of capillary coverage was interpreted as the amount of biofilm formed, which was calculated using the Bioflux Montage software. Yellow scale bars for photographic panels **(E)** are equal to 20 µm. In all cases, values are from three biological replicates (n = 3); the results are the mean ± standard deviations. Statistical analysis was performed using the Kruskal–Wallis test with Holm correction. p < 0.05 was considered statistically significant (*), while *p* > 0.05 but < 0.1 was considered statistical tendency (^#^).

In the next set of studies, we investigated how ¼× MIC antibiotic values affect biofilm development under more dynamic, microfluidic conditions generated by the Bioflux 1000 system. A microfluidic model of biofilm development by clinical *H. pylori* strains, including those used in the current studies, was validated and published by us previously (Krzyżek et al., 2022) (Figures 6A–C).

We determined that under microfluidic settings a constant exposure of bacteria to ¼× MIC of antibiotics was accompanied by a reduction in the biofilm formation in all of the analyzed scenarios (each strain and each antibiotic) relative to the control (Figures 6D,E). Despite this, such an effect was only significant when bacteria were exposed to ¼× MIC of CLR, contributing to the development of approximately 40% of the biofilm observed in the control. When bacteria were exposed to ¼× MIC of MTZ or LEV, the amount of biofilm formed was on the average level of 70%–80% of that recorded in the control, while this difference was not statistically significant.

The above observations show the existence of certain discrepancies between the results obtained in the classical, static conditions and those observed in the microfluidic model. We presume that under conditions of a constant medium flow, the autoaggregation of the tested *H. pylori* strains is strongly limited. This seems to be associated with the fact that single cells or small aggregates that can attach to each other and build up larger bacterial clusters during stationary culture conditions are often mechanically removed in microfluidic systems (Béchon et al., 2020; Krzyżek et al., 2022). Therefore, microfluidic experiments presented above uncover that the process of biomatrix production during environmental stress caused by the constant antibiotic exposure, despite its importance, does not seem to fully compensate for the protection caused by the rapid and intensive autoaggregation of *H. pylori*. In the research of Burel et al. (2021), using *Staphylococcus aureus*, it was shown that autoaggregation plays an important role in tolerance to antimicrobial substances. The team determined not only that the autoaggregation was a highly dynamic process (occurring over minutes), but more importantly that it resulted from changes in bacterial membrane properties rather than the secretion of matrix components.

### 2.6 Antibiotics modify the membrane fatty acid profile of *H. pylori*

Inspired by the above data, in the last set of experiments, we determined how antibiotic exposure of the tested *H. pylori* strains affects their cell membrane lipid profile. Cell membranes are complex and dynamic structures, composed mainly of lipids (Ernst et al., 2016). To preserve membrane stability, microorganisms must continuously adapt and modify the fatty acid composition of membranes in response to a variety of environmental stimuli. This process is often referred to as homeoviscous adaptation and is crucial for maintaining optimal fluidity and permeability of membranes, and thus microbial survival (Ernst et al., 2016; May and Grabowicz, 2018). It is indicated that this phenomenon is of great importance in limiting the access of antibiotics to their target sites in bacterial cells (May and Grabowicz, 2018). Although the pattern of fatty acids building up cell membranes of *H. pylori* was investigated over three decades ago (Inamoto et al., 1993), just recently the use of modern analytic techniques allowed scientists to precisely determine the fatty acid profile of this bacterium (Nagata et al., 2021). Using the data on the most important fatty acids constituting *H. pylori* cell membranes (Inamoto et al., 1993; Nagata et al., 2021), we decided to fill the knowledge gap and demonstrate how this profile changes when bacteria are exposed to sub-MICs of antibiotics.

**FIGURE 7:**
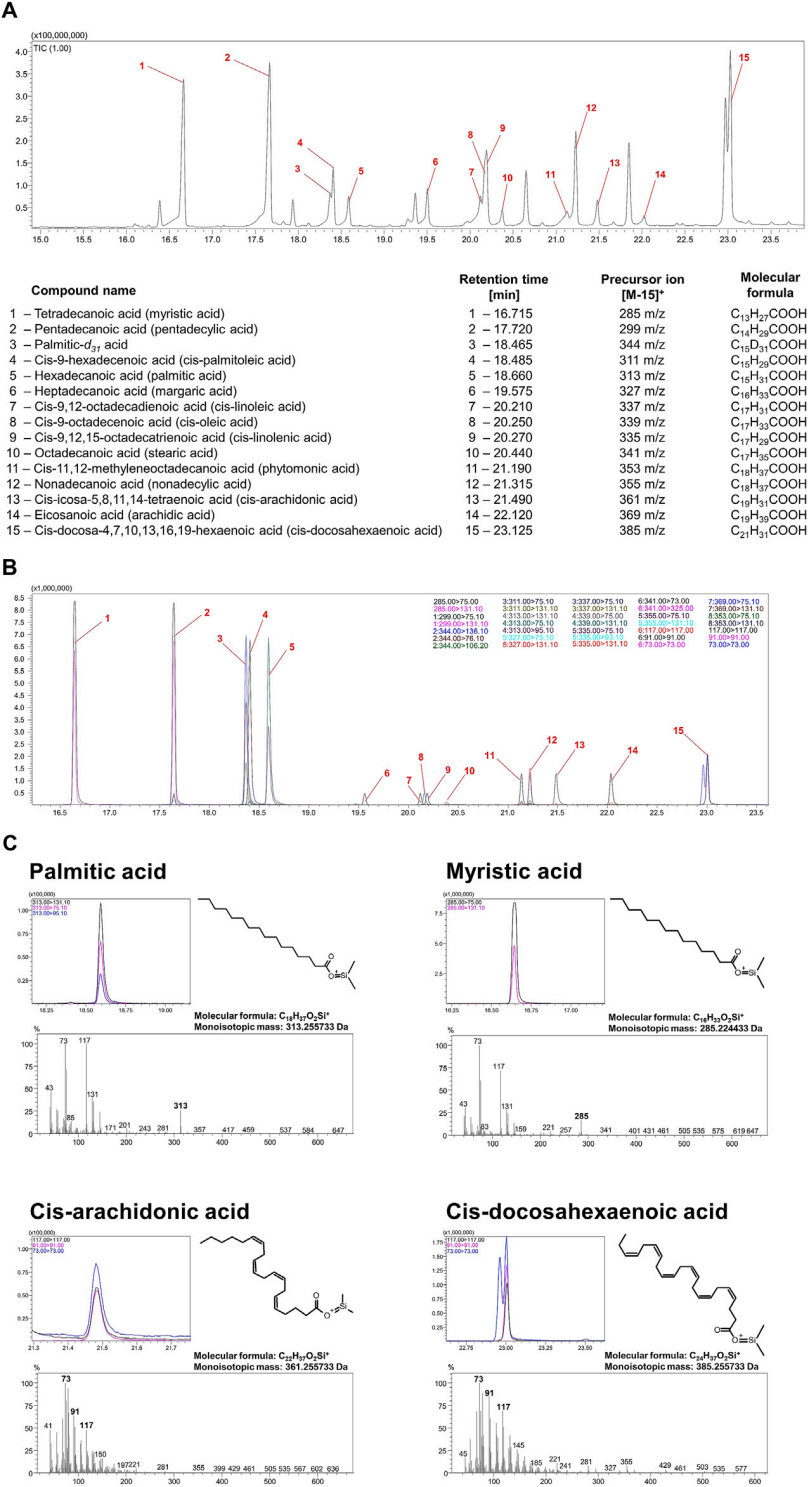
Validation of the methodology determining the most important fatty acids constituting cell membranes *H. pylori*. Chromatographic spectra recorded in scan **(A)** and MRM **(B)** mode of the analytical standards of all fatty acids analyzed. **(C)** Chromatographic spectra in MRM mode and mass spectrometry spectra in scan mode of analytical standards of four selected fatty acids: myristic acid (C14:0), palmitic acid (C16:0), cis-arachidonic acid (C20:4) and cis-docosahexaenoic acid (C22:6).

Bacterial lipidomic analyses are complicated, but yet increasingly developed, due to the sparse availability of data covering this topic. The results obtained, however, have a very high scientific potential and shed more light on such topics as microbial identification or antibiotic resistance (Li et al., 2010). Classically, omics analyses, including lipidomics of bacteria, can be divided into targeted and untargeted (Appala et al., 2020). Our research team decided to use targeted (based on analytical standards) lipidomic analysis of bacteria using a gas chromatography coupled with tandem mass spectrometry (GC-QqQ-MS/MS). This technique allows for the determination of only selected fatty acids (those for which analytical standards have been purchased), although in contrary to untargeted lipidomic analysis it permits to optimize the conditions for their determination (including the selection of the optimal extraction method from the matrix, chromatographic conditions, and the conditions and mode of operation of the mass spectrometer). All of this translates directly into an increase in selectivity, specificity and sensitivity of determinations and thus enables to obtain more reliable results. In the method described in the current paper, the choice of appropriate extraction, sample preparation, chromatographic conditions and mass spectrometer parameters have been optimized (Figure 7).

**FIGURE 8:**
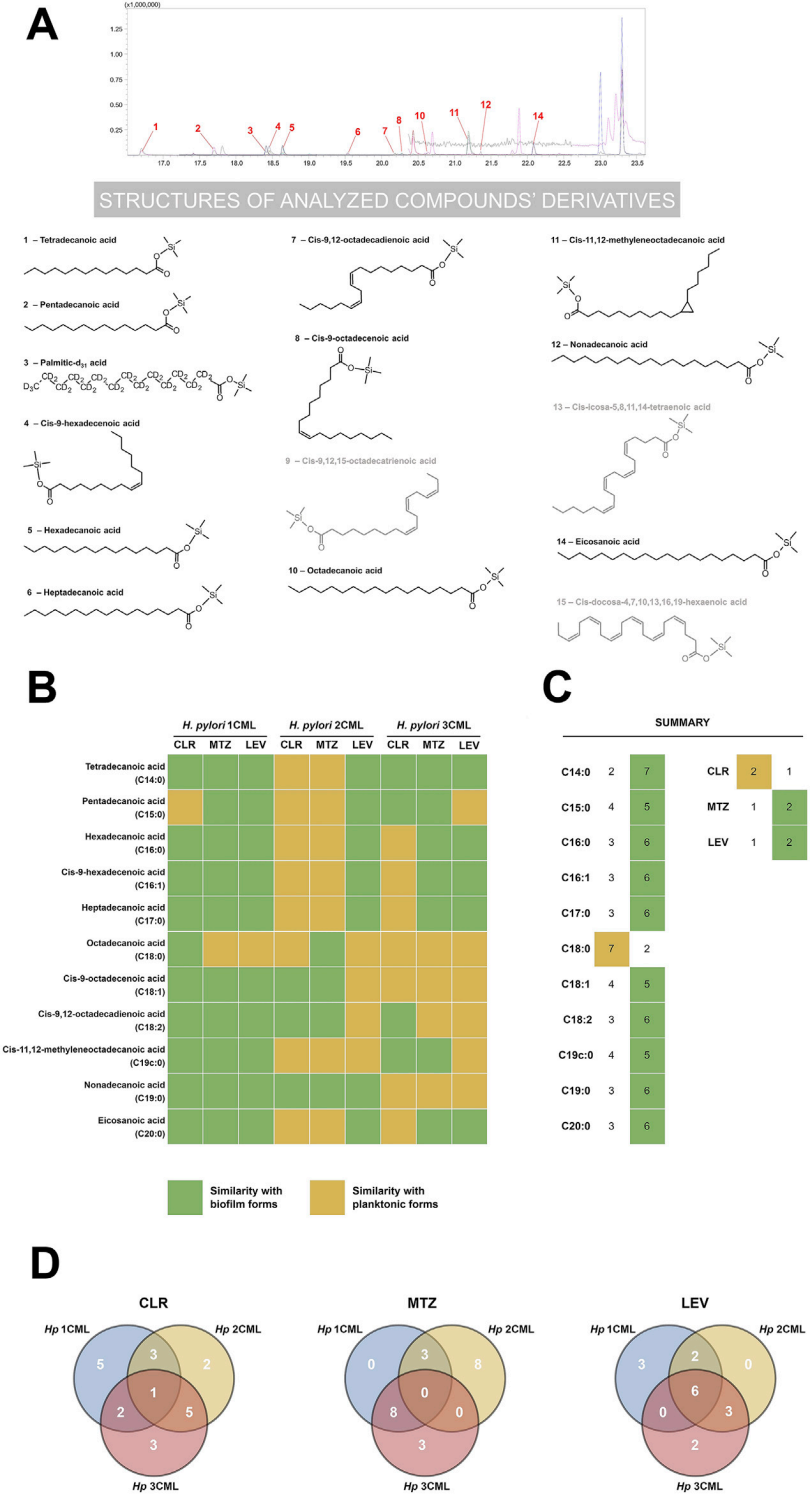
The effect of antibiotics on the fatty acid profile of cell membranes of *H. pylori*. Chromatographic spectrum in MRM mode of an exemplary bacterial sample **(A)** with structural formulae of all analyzed fatty acids in the form of their derivatives (fatty acids present in the sample are shown in black, those absent in the sample in gray). Heat map **(B)** showing the effect of ¼× MIC of antibiotics (clarithromycin [CLR], metronidazole [MTZ] and levofloxacin [LEV]) on the fatty acid profile of *H. pylori* strains. Quantitative changes in each of the analyzed fatty acids were compared with control samples (native planktonic cells and native biofilm cells). On this basis, values closer to the data obtained for planktonic and biofilm forms were categorized qualitatively as “similarity with planktonic cells” and “similarity with biofilm cells”, respectively. Bacteria were cultured in 12-well microtiter plates for 3 days at 37°C, microaerophilic conditions and shaking at 100 rpm (experimental and control planktonic cells) or 50 rpm (control biofilm cells). The data obtained from the analysis of point **B** were summed and presented as point **(C)**. Venn diagram **(D)** showing the specifically or commonly modified cell membrane fatty acids of the tested *H. pylori* strains in response to ¼× MIC of antibiotics.

Using untargeted analysis can pose many difficulties in identifying lipids with similar chemical structures whose mass spectra are very similar to each other, as can be seen in Figure 7C, which depicts the mass spectra of palmitic acid, myristic acid, cis-arachidonic acid and cis-docosahexaenoic acid. While the precursor ions [M-15]^+^ for palmitic acid and myristic acid have a satisfactory abundance and the spectra for them are quite characteristic to identify these acids even in an untargeted analysis, we did not observe precursor ions in the case of cis-arachidonic acid and cis-docosahexaenoic acid. This example demonstrates that despite the distinct differences in the chemical structure of unsaturated acids in particular, the mass spectra that characterize them are very similar, and therefore the identification of these compounds using the available databases without performing a comparative analysis of the relevant analytical standards may lead to an incorrect interpretation. Similar observations have been made by other authors studying fatty acids (Beccaria et al., 2018; Wheelan et al., 1995), so many interesting approaches to the analysis of this group of compounds can be found when no analytical standards are available, e.g., reducing the ionization energy to as low as 20 eV in an electron impact mass spectrometer (EI-MS) compared to the standard 70 eV or using double derivatization. To the best of our knowledge, the most reliable results are provided by targeted analysis in multiple reaction monitoring (MRM) mode based on commercial analytical standards for fatty acids and therefore in the current article we applied this type of approach (Figure 7; Figure 8A).

Using a comparative analysis (planktonic forms treated with antibiotics vs. native planktonic and biofilm forms), in the first stage of the current examinations, we determined how antibiotic exposure of *H. pylori* strains affects the quantity of the analyzed membrane fatty acids (Figures 8B,C). We noticed that in bacteria treated with sub-MICs of antibiotics the fatty acid profile resembled the phenotype of biofilm forms (10 out of the 11 examined fatty acids). Only in the case of octadecanoic acid (C18:0), we did not observe any significant modifications after treating bacteria with antibiotics (similarity with planktonic forms). Although difficult to clearly justify, we suspect that derivatives of this fatty acid (cis-9-octadecenoic acid [C18:1], cis-9,12-octadecadienoic acid [C18:2] and cis-11,12-methyleneoctadecanoic acid [C19c:0]), which are abundant in *H. pylori* membranes under native conditions (Inamoto et al., 1993; Nagata et al., 2021), may play a more important role in the survival of this bacterium. The most uniform example in our experiments were bacteria exposed to ¼× MIC of LEV, as we noticed an increase in the ratio of C19c:0/C18:0 in all three *H. pylori* strains (Supplementary Table S1). In this context, it is worth re-calling the research results of Jiang et al. (2019), who discovered that C19c:0 affects a number of pathogenicity traits of *H. pylori*, including tolerance to antibiotics. Therefore, future research should focus on confirming the hypothesis about the importance of C19c:0 in the biofilm development and generation of antibiotic resistance.

In the next stage of the comparative analysis, we assessed separately the impact of each of the tested antibiotics on the overall nature of changes in the cell membrane fatty acid profile (Figures 8B,C). We managed to confirm our previous observations indicating that bacteria exposed to ¼× MIC of CLR resemble more planktonic forms, while bacteria treated with ¼× MIC of MTZ or LEV presented a biofilm-like phenotype (Figures 2–5). This suggests that sub-MICs of antibiotics have a global effect on the physiology of *H. pylori* that affects not only the secretion of extracellular matrix (Figure 2), but also modify the cell membrane profile (Figure 8). The above changes may consequently modulate the ability of *H. pylori* to form biofilm.

In the last stage, we decided to estimate the inter-strain degree of similarity in the modification of cell membrane fatty acids when bacteria were exposed to a specific antibiotic (Figure 8D). We discovered the highest degree of similarity between *H. pylori* 2CML and 3CML (two of the three conditions tested), which is consistent with their high similarity in the autoaggregation rate (Figure 3). Interestingly, however, we did not note any similarities after treating these strains with ¼× MIC of MTZ. Moreover, by examining response patterns of all *H. pylori* strains, we observed high discrepancies in their fatty acid profiles (Figure 8D). The exception was bacteria exposed to ¼× MIC of LEV as in all three strains 6 out of the 11 fatty acids underwent similar changes. We believe that the above situation, related to the lack of uniform changes in the membrane fatty acids in response to antibiotics, is strongly related to the high heterogeneity between isolates of this bacterium. In this regard, many scientists in the discussion of different *H. pylori* strains often use the term “quasi-species“ (Kuipers et al., 2000). This issue is undoubtedly further complicated by the high heterogeneity of microbial biofilm cells themselves (Karygianni et al., 2020). All of this translates potentially to the situation in which future attempts to find molecular targets of anti-biofilm therapies active against a wide range of different *H. pylori* strains may prove to be much more difficult than initially expected. Undoubtedly, further research in this area is necessary.

### 2.7 Limitations of the study and future perspectives

Although we believe that the current study may significantly deepen the state of knowledge regarding the modulatory effect of antibiotics on the biofilm development of *H. pylori*, we would like to point out some limitations of our research.

Firstly, our experiments were focused on three out of the five classically used antibiotics, such as CLR, MTZ and LEV. This choice was dictated by the data on the global antibiotic resistance of *H. pylori*, where the current level of resistance against these three has exceeded the acceptable threshold of 15% (Savoldi et al., 2018; Yu et al., 2024). Keeping in mind both the above observations and the data suggesting strong implications of biofilm formation in the development of antimicrobial resistance in different pathogens (Shree et al., 2023; Uruén et al., 2020), we decided to narrow our studies to antibiotics against which *H. pylori* is characterized by a high epidemiological level of resistance. The current study did not verify the effects of amoxicillin and tetracycline against *H. pylori* as the level of resistance to these two antibiotics is marginal in most regions of the world (Savoldi et al., 2018; Yu et al., 2024). Nevertheless, at this point, we cannot reject the possibility that they can also modulate the biofilm-forming properties of this bacterium.

Secondly, we based our observations of the current research only on the results of *in vitro* experiments. We realize that a simple translation of the data obtained in laboratory conditions into a real-life scenario is burdened with some limitations. Therefore, in order to increase the credibility of our laboratory studies, the observations made herein included a number of experimental techniques (culture, fluorescence microscopy with a set of various post-microscopical analyses, microfluidics, and targeted lipidomics) that enabled for a more robust verification of the hypothesis. In spit of that, for the purpose of increasing the reliability of the observations, our future plans will seek to confirm the results obtained in the current article in more complex research models, including higher organisms.

## 3 Materials and methods

### 3.1 Bacterial strains

For the purpose of the current research, we used three clinical strains of *H. pylori* (1CML, 2CML, 3CML) belonging to the collection of the Department of Microbiology, Wroclaw Medical University in Poland, which in a previous study of our team were categorized as the strongest biofilm producers (Krzyżek et al., 2022). Based on the EUCAST clasiffication (2024), all three strains were classified as multidrug-resistant, presenting resistance to CLR (>0.25 μg/mL), MTZ (>8 μg/mL) and LEV (>1 μg/mL) in solid agar cultures and an E-test-based sensitivity determination (Krzyżek et al., 2022). The strains were stored frozen at −80°C in Tryptic-Soy Broth (TSB; Oxoid, Dardilly, France) supplemented with 30% glycerol until tests were performed. They were revived by sowing on Columbia agars (Difco, Lublin, Poland) with 10% horse blood (Graso Biotech, Starogard. Gdański, Poland) and incubation for 3 days at 37°C and a microaerophilic atmosphere (Genbox microaer kits; BioMerieux, Marcy I'Etoile, France). After this time, bacteria were passaged once onto new solid media and cultured again under the above conditions to obtain the appropriate viability of bacterial cells.

### 3.2 Influence on the viability of planktonic and biofilm forms

#### 3.2.1 Determination of MIC and MBC values in liquid cultures

Determination of MICs and MBCs of *H. pylori* strains against CLR, MTZ and LEV (all from Sigma-Aldrich, St. Louis, MO, United States) was performed using a broth microdilution method and a spotting method, respectively (Krzyżek et al., 2021). Cultures were performed in 12-well microtiter plates (Bionovo, Legnica, Poland) filled in each well with 1 mL of bacterial suspension (10^8^ CFU/mL) in Brain-Heart Infusion broth (BHI; Oxoid, Dardilly, France) + 5% fetal calf serum (FCS; Gibco, Paisley, Scotland, United Kingdom) and a concentration gradient of one of the tested antibiotics (1–512 μg/mL). A well containing bacterial suspension in BHI + 5% FCS without antibiotic and pure culture medium without bacteria served as positive and negative controls, respectively. Microtiter plates were cultured for 3 days at 37°C, microaerophilic conditions and shaking at 100 rpm (MaxQ 6000, Thermo Fisher, Waltham, MA, United States). The well containing the lowest antibiotic concentration in which lack of turbidity was observed was defined as the MIC. Then, 10 µL of the suspension was taken from each well of the microtiter plate and spotted onto Columbia agars with 10% horse blood. The plates were incubated for 3 days at 37°C and microaerophilic conditions. The spot with the lowest antibiotic concentration in which no bacterial colony growth was observed was classified as the MBC. The tests were performed in six biological replications (n = 6/strain).

#### 3.2.2 Viability of planktonic forms

The viability of planktonic *H. pylori* forms exposed to a concentration gradient of each of the tested antibiotics (¼× MIC – 4× MIC) was determined by culture in microtiter plates and assessment of the bacterial optical density (Chen et al., 2023). The control consisted of bacteria not exposed to any antibiotic. The 12-well microtiter plates filled in each well with 1 mL of bacterial suspension (10^8^ CFU/mL) in BHI + 5% FCS and a given concentration of antibiotic were incubated for 3 days at 37°C, microaerophilic conditions and shaking at 100 rpm. After this period, 200 µL of bacterial suspension was collected from each well and transferred to 96-well microtiter plates (Bionovo, Legnica, Poland). Bacterial viability was assessed by measuring optical density at a wavelength of 600 nm (OD_600_) using an Asys UVM 340 microplate reader (Biochrom Ltd., Cambridge, United Kingdom). The absorbance of the negative control (pure culture medium) was subtracted from the absorbance of each tested sample. The tests were performed in three biological replications with three technical repetitions (n = 9/strain).

#### 3.2.3 Viability of biofilm forms

The viability of biofilm *H. pylori* forms exposed to a concentration gradient of each of the tested antibiotics (¼× MIC – 4× MIC) was assessed by culture in microtiter plates and determination of the number of colonies grown from mechanical removal of the biofilm (Grande et al., 2021). The control consisted of bacteria not exposed to any antibiotic. For this purpose, 12-well microtiter plates were filled in each well with 1 mL of bacterial suspension (10^8^ CFU/mL) in BHI + 5% FCS and a given concentration of antibiotic. The plates were incubated for 3 days at 37°C, microaerophilic conditions and shaking at 50 rpm. After this incubation period, the entire bacterial suspensions were removed from each well and the wells were rinsed once with a phosphate buffer solution (PBS; Sigma-Aldrich, St. Louis, MO, United States) to remove unadhered bacterial cells. The wells were filled again with 1 mL of BHI + 5% FCS, and then bacteria adhered to the surface of the well walls were mechanically scraped off using Corning cell scrapers (Sigma-Aldrich, St. Louis, MO, United States). The entire volume of the suspensions were transferred to sterile Eppendorf tubes (Bionovo, Legnica, Poland) and several dilutions were made in BHI + 5% FCS broth. A 100 µL of each dilution was taken and inoculated on the surface of Columbia agars with 10% horse blood. After 3 days of incubation at 37°C and microaerophilic conditions, the number of grown colonies was counted. The tests were performed in three biological replications with three technical repetitions (n = 9/strain).

### 3.3 Influence on the proteins amount

The impact of ¼× MIC of each tested antibiotic on the amount of proteins in bacterial cells was determined using a Rapid Gold Pierce BCA protein assay (ThermoFisher, Waltham, MA, USA) and a spectrophotometric quantification. The experimental control consisted of bacteria not exposed to any antibiotic. To obtain bacterial biomass, 12-well microtiter plates filled in each well with 1 mL of bacterial suspension (10^8^ CFU/mL) in BHI + 5% FCS and a given antibiotic concentration were incubated for 3 days at 37°C, microaerophilic conditions and shaking at 100 rpm. Then, bacterial suspensions were transferred to Eppendorf tubes and subjected to centrifugation at 8,000 rpm for 5 min (Gusto High-Speed Mini Centrifuge; Heathrow Scientific LLC, Vernon Hills, IL, United States). The supernatant was discarded from each sample, bacterial pellets were rinsed with 1 mL of PBS and subjected for the centrifugation once again. Supernatant was removed and Eppendorf tubes with bacterial pellets were dried for 10 min at 60°C (Thermo-shaker; BioSan, Riga, Latvia). Samples were weighed (Radwag, Radom, Poland) to obtain information about the dry bacterial biomass. After this step, 0.2 mL of RIPA Lysis and Extraction Buffer (ThermoFisher, Waltham, MA, United States) was added to the bacterial pellets and the samples were shaken for 15 min at room temperature (Vortex Mixer; Velp Scientifica, Usmate Velate, Italy). After obtaining bacterial lysates, the samples were directed to a series of steps related to measuring the amount of proteins. Briefly, following the recommendations of the manufacturer, reagent A and B were mixed together in a 50:1 ratio to obtain the BCA working solution. A 20 µL of bacterial lysate solution and 200 µL of BCA working solution were added to each well of a 96-well microtiter plate, and the plate was incubated for 5 min at room temperature. Absorbance of the samples was measured at a wavelength of 480 nm (OD_480_) using an Asys UVM 340 microplate reader. The concentration of the tested samples was determined on the basis of the albumin absorbance curve (0–1 mg/mL). The obtained results were normalized to the weight of the dry bacterial biomass. The tests were performed in three biological replications with three technical repetitions (n = 9/strain).

### 3.4 Influence on the oxidative stress level

The impact of ¼× MIC of each tested antibiotic on the oxidative stress level in bacterial cells was determined using a H2DCFDA staining (ThermoFisher, Waltham, MA, United States) and fluorescence estimation. The experimental control consisted of bacteria not exposed to any antibiotic. To obtain bacterial biomass, 12-well microtiter plates filled in each well with 1 mL of bacterial suspension (10^8^ CFU/mL) in BHI + 5% FCS and a given antibiotic concentration were incubated for 3 days at 37°C, microaerophilic conditions and shaking at 100 rpm. The manufacturer’s instructions were followed in the next steps. Briefly, 0.5 mL of bacterial suspensions were transferred to Eppendorf tubes and subjected to centrifugation at 8,000 rpm for 5 min. The supernatant was discarded from each sample, bacterial pellets were rinsed with 1 mL of PBS and subjected for the centrifugation once again. A 0.1 mL of solution with 10 μg/mL H2DCFDA in PBS was added to each Eppendorf tube. The samples were incubated for 15 min at room temperature. After this step, 20 µL of each bacterial suspension was spotted on glass slides and directed for microscopic observations. The level of oxidative stress was calculated based on the fluorescence intensity of bacterial cells. Pictures were taken using a Carl Zeiss inverted fluorescence microscope (GmbH, Jena, Germany) and the fluorescence level was determined using a Bioflux Montage software (Fluxion, San Francisco, CA, United States). The tests were performed in three biological replications with three technical repetitions constituting different observation fields of the examined glass slide (n = 9/strain).

### 3.5 Effect on the biochemical parameters

The impact of selected concentrations (¼× MIC and MIC) of each of the tested antibiotics on the biochemical parameters of *H. pylori* biofilm was assessed by multiple, selective fluorescent staining and determination of their fluorescence intensity and spatial co-localization (Krzyżek et al., 2022). The control consisted of bacteria not exposed to any antibiotic. For this purpose, 12-well microtiter plates filled in each well with 1 mL of bacterial suspension (10^8^ CFU/mL) in BHI + 5% FCS and a given antibiotic concentration were incubated for 3 days at 37°C, microaerophilic conditions and shaking at 50 rpm. Then, the bacterial suspension was removed from each well, gently rinsed with 1 mL of PBS, and then refilled with a solution containing fluorescent dyes staining the selective biofilm components: 0.3 µL of SYTO9 (ThermoFisher, Waltham, MA, United States), 1 µL of DAPI (ThermoFisher, Waltham, MA, United States) and 100 µL of SYPRO RUBY (ThermoFisher, Waltham, MA, United States), which enable the assessment of the amount of bacterial biomass, extracellular DNA and extracellular proteins. The plate was left for a 15 min-incubation in the dark and then observed using a Carl Zeiss inverted fluorescence microscope. Both the fluorescence intensity of individual biofilm components and their co-localization (eDNA/biomass, proteins/biomass, eDNA/proteins and proteins/eDNA) were calculated using the Bioflux Montage software. The tests were performed in three biological replications with five technical repetitions constituting different observation fields of the examined well (n = 15/strain).

### 3.6 Effect on the autoaggregation and biophysical parameters

The impact of selected concentrations (¼× MIC and MIC) of each of the tested antibiotics on the rate and intensity of autoaggregation was assessed using a time-lapse live analysis observation with an inverted fluorescence microscope and a series of post-microscopic computer analyzes (Krzyżek et al., 2022). The experimental control consisted of bacteria not exposed to any antibiotic.

The wells of 12-well microtiter plates were filled with 1 mL of high-density bacterial suspensions (10^9^ CFU/mL) to maximize the rate of autoaggregation. Bacterial suspensions were prepared in 1 mL of BHI + 5% FCS and a given antibiotic concentration. To facilitate better visualization of bacteria, each well was additionally loaded with 1 μL/mL of the non-toxic fluorescent dye from the CellTrace CFSE Cell Proliferation Kit (ThermoFisher, Waltham, MA, United States). Bacterial cells were incubated for 10 min at 37°C to allow the dye to be absorbed. Microtiter plates with bacterial suspensions were placed in an environmental incubator (Pecon Incubator XL S1, Carl Zeiss, Jena, Germany) combined with an inverted fluorescence microscope at 37°C and microaerophilic conditions. Photographs of bacteria were taken automatically every 1 min for a period of 1 h using the Bioflux Montage software. After completing the series of experiments, the rate of coverage of the observation field was counted and interpreted as the speed of autoaggregation. Autoaggregation at a given time point (T_1_ – T_60_) was calculated by subtracting the autoaggregation at the initial point of the experiment (T_0_). The tests were performed in three biological replications with three technical repetitions constituting different observation fields of the examined well (n = 9/strain).

In the next step, the total number of bacterial aggregates of a given size was calculated from photographs showing the results of 1-h autoaggregation. At the very beginning, the size of the aggregates was normalized to dimensions of the observation field in the examined photo. Bacterial clusters with sizes > 1%, 1%–0.5%, 0.5%–0.1% and < 0.1% of the observation field were classified as large, big, medium and small, respectively (Krzyżek et al., 2022). Readings with values smaller than the size of a single spherical *H. pylori* form were recorded as artifacts and discarded from the total pool of results. After categorizing all the aggregates in the analyzed photo, the total area occupied by the aggregates of a given category was calculated. This data was presented as a percentage share of aggregates of a specific category. The tests were performed in three biological replications with three technical repetitions constituting different observation fields of the examined well (n = 9/strain).

Additionally, immediately after completing the autoaggregation experiments, a Z-stacking of the tested samples was performed (Chun et al., 2022; Marra et al., 2023). Using a Carl Zeiss inverted fluorescence microscope and the Bioflux Montage software, a total of 50 Z-stacks were collected in each case from the center of the examined well of the microtiter plate. All Z-stacks were performed at an interval of 1 µm. After obtaining images, the Z-stacks were processed using ImarisViewer software (Oxford Instruments, Abingdon, United Kingdom) to generate three-dimensional projections of bacterial biofilms. The tests were performed in three biological replications (n = 3/strain).

### 3.7 Effect on the ultrastructure of aggregates

The ultrastructure of 1-h aggregates exposed to ¼× MIC of each tested antibiotic was determined using scanning electron microscopy (Krzyżek et al., 2022). The experimental control consisted of bacteria not exposed to any antibiotic. To obtain aggregates, high-density bacterial suspensions (10^9^ CFU/mL) were prepared in 12-well microtiter plates filled in each well with 1 mL of BHI + 5% FCS and a given antibiotic concentration. Prepared suspensions were directed for a 1-h incubation at 37°C and microaerophilic conditions. After this time, bacterial suspensions containing the aggregates were transferred to Eppendorf tubes and subjected to centrifugation at 8,000 rpm for 5 min. The supernatant was discarded from each sample and bacterial pellets obtained in this way were sent for a set of fixation steps. The tests were performed in three biological replications (n = 3/strain).

Pelleted bacterial aggregates were immersed in 0.5 mL of 2.5% glutaraldehyde solution (Sigma-Aldrich, St. Louis, MO, United States) and incubated for 1 day at 4°C. After this step, the tested samples were rinsed three times in 0.1 M cacodylate solution (Sigma-Aldrich, St. Louis, MO, United States). Then, the samples were passed through an increasing alcohol gradient (30%, 50%, 70%, 90%, and 99.8%). Finally, the fixed samples were dusted with a layer of carbon using an EM ACE600 sputter (Leica Microsystems, Wetzlar, Germany) and observed using an Auriga 60 scanning electron microscope (Oberkochen, Germany).

### 3.8 Effect on biofilm formation in microfluidic conditions

Biofilm formation in microfluidic conditions was carried out using the Bioflux1000 system (Fluxion, San Francisco, CA, United States), dedicated 48-well microfluidic plates (equipped with 24 inlet and outlet wells connected by microcapillaries) (Fluxion, San Francisco, CA, United States), and a Pecon environmental chamber maintaining appropriate culture conditions. The procedure for biofilm development by *H. pylori* using the Bioflux1000 system has been validated by us in the previous study (Krzyżek et al., 2022). In the already developed version of the experiment, the microcapillaries of the plate were rinsed with a strong stream of 0.1 mL BHI + 5% FCS at an intensity of 10 dyne/cm^2^ for 10 s. After unblocking the channels, the culture medium was left for 15 min of incubation to enable the components to precoat the walls of microcapillaries. In the next step, the inlet wells were emptied and refilled with 1 mL of bacterial suspension (10^8^ CFU/mL) in BHI + 5% FCS and ¼× MIC of one of the tested antibiotics. The study control consisted of bacteria not exposed to any antibiotic. Then, the medium flow was turned on from the inlet to the outlet direction at a rate of 0.1 dyne/cm^2^ for 24 h at 37°C and microaerophilic conditions. After 1 day of incubation, the flow was stopped and photographs of the biofilms formed in the microcapillaries were taken using a Carl Zeiss inverted microscope. The degree of capillary coverage was interpreted as the amount of biofilm formed, which was calculated using the Bioflux Montage software (Fluxion, San Francisco, CA, United States). The tests were performed in three biological replications (n = 3/strain).

### 3.9 Targeted lipidomic analysis of bacteria

#### 3.9.1 Chemicals

Isooctane, water (Chromasolv^®^ LC–MS) and methanol (Chromasolv^®^ LC–MS) were purchased from Sigma-Aldrich (Steinheim, Germany); Analytical standards of fatty acids: tetradecanoic acid (1,000 μg/mL in isooctane), pentadecanoic acid (1,000 μg/mL in isooctane), cis-9-hexadecenoic acid (1,000 μg/mL in isooctane), hexadecenoic acid (1,000 μg/mL in isooctane), heptadecanoic acid (1,000 μg/mL in isooctane), cis-9,12-octadecadienoic acid (1,000 μg/mL in isooctane), cis-9-octadecenoic acid (1,000 μg/mL in isooctane), cis-9,12,15-octadecatrienoic acid (1,000 μg/mL in isooctane), octadecanoic acid (1,000 μg/mL in isooctane), nonadecanoic acid (1,000 μg/mL in isooctane), cis-icosa-5,8,11,14-tetraenoic acid (1,000 μg/mL in isooctane), eicosanoic acid (1,000 μg/mL in isooctane) were purchased from Chiron (Trondheim, Norway), cis-docosa-4,7,10,13,16,19-hexaenoic acid (10 mg as neat powder) and hexadecanoic-*d*_31_ acid (25 mg as neat powder) were purchased from Sigma-Aldrich (Steinheim, Germany); cis-11,12-methyleneoctadecanoic acid (10 mg/mL in ethanol) was purchased from Larodan AB (Solna Sweden); *N*-methyl-*N*-(trimethylsilyl)trifluoroacetamide was purchased from Sigma-Aldrich (St. Louis, United States). The analytical standard in powder form was firstly dissolved in isooctane. Standard solutions of fatty acids and internal standard (IS) palmitic-*d*_31_ acid were then prepared in isooctane at concentration of 100 μg/mL and 10 μg/mL, respectively. The stock solutions and standard solutions were stored at −20°C.

#### 3.9.2 Sample preparation

To obtain bacterial biomass, 12-well microtiter plates filled in each well with 1 mL of bacterial suspension (10^8^ CFU/mL) in BHI + 5% FCS and ¼× MIC of one of the tested antibiotics were incubated for 3 days at 37°C, microaerophilic conditions and shaking at 100 rpm (experimental and control planktonic cells) or 50 rpm (control biofilm cells). The experimental controls consisted of non-adhered bacteria not exposed to any antibiotic (native planktonic cells) and well-adhered bacteria not exposed to any antibiotic (native biofilm cells).

Bacterial cells from experimental samples and native planktonic cells were obtained directly from the culture suspensions. Native biofilm cells were obtained by removing the entire bacterial suspensions and scraping bacterial biomass from the walls of the titration plate re-filled again with 1 mL of BHI + 5% FCS. The optical density of all samples was normalized to 1 McFarland unit (densitometer; BioSan, Riga, Latvia) by adding an appropriate volume of pure BHI + 5% FCS, and 1 mL of such a sample was directed to further stages of obtaining bacterial biomass. Bacterial suspensions were transferred to Eppendorf tubes and subjected to centrifugation at 8,000 rpm for 5 min. The supernatant was discarded from each sample, bacterial pellets were rinsed with 1 mL of PBS and subjected for the centrifugation once again. Supernatant was removed and Eppendorf tubes with bacterial pellets were directed to a series of steps related to the isolation of fatty acids.

In this regard, 50 µL of water was added to Eppendorf tubes with bacterial pellets. After vortex-mixing for 5 s, 10 µL of IS solution (palmitic-d31 acid, 10 μg/mL in isooctane) was added. Next, liquid-liquid extraction (LLE) with 0.5 mL of isooctane was carried out for 30 min in an ultrasound bath. The sample was then centrifuged at 13,500 rpm for 3 min. The organic phase was transferred into 2-mL Eppendorf tubes and evaporated to dryness under a stream of inert nitrogen gas at 40°C. The dry residues were dissolved in 50 µL of N-methyl-N-(trimethylsilyl)trifluoroacetamide solution and the tubes were then heated at 70°C for 20 min. After cooling, the solution was transferred into glass inserts of autosampler vials and analyzed by GC-QqQ-MS/MS. The tests were performed in three biological replications (n = 3/strain).

#### 3.9.3 Instrumentation

Analyses were performed using a gas chromatograph (GC, Shimadzu QP 2010; Kyoto, Japan) operated with an autosampler AOC-20s and autoinjector AOC-20i (Shimadzu, Milan, Italy). The separation was done using a SH-RXI-5MS column (30 m × 0.25 mm i.d., particle size 0.25 µm; Shimadzu, Bellefonte, Pennsylvania, United States). The column temperature was initially held at 60°C for 2 min, increased at 10°C/min to 320°C and held there for 15 min. Helium (purity 6.0, Messer, Gumpoldskirchen, Austria) was used as a carrier gas at a flow-rate of 1.56 mL/min. The syringe size was 10 µL. The injection volume was 0.5 μL. A splitless injection mode was applied with sampling time of 1.0 min. Injector temperature was 260°C. The injector was set to auto cleaning by pre-injecting ethyl acetate and acetonitrile. Total run time was 43 min.

Detection of fatty acids was achieved using a triple-quadrupole mass spectrometer (QqQ, Shimadzu TQ8040, Kyoto, Japan). The spectrometer was equipped with an electron ionisation (EI) source; determination of fatty acids was carried out in the multiple reaction monitoring (MRM) mode. The following MS parameters were fixed: ion source temperature, 200°C; interface temperature, 280°C; electron ionization energy was 70 eV; the detector voltage was set at 1.0 kV. A summary of precursor and product ions, collision energies, loop time, and retention time for each compound is presented in Table 1.

**TABLE 1:** MRM conditions used in the GC-QqQ-MS/MS method for quantification of fatty acids in bacteria.

Compound	Precursor ion [m/z]	Product ion [m/z]	Loop time [sec]	Collision energy [V]	Retention time [min]
Tetradecanoic acid (myristic acid)	285.0	75.0* 131.1	0.3	18.0 9.0	16.715
Pentadecanoic acid (pentadecylic acid)	299.0	75.1* 131.1	0.3	15.0 9.0	17.720
Hexadecanoic-*d*_*31*_ acid (palmitic-*d*_*31*_ acid)	344.0	136.1* 106.2 76.1	0.3	6.0 6.0 6.0	18.465
Cis-9-hexadenenoic acid (cis-palmitoleic acid)	311.0	75.1* 131.1	0.3	15.0 12.0	18.485
Hexadecanoic acid (palmitic acid)	313.0	131.1* 95.1 75.1	0.3	6.0 6.0 6.0	18.660
Heptadecanoic acid (margaric acid)	327.0	75.1* 131.1	0.3	12.0 12.0	19.575
Cis-9,12-octadecadienoic acid (cis-linoleic acid)	337.0	75.1* 131.1	0.3	15.0 12.0	20.210
Cis-9-octadecenoic acid (cis-oleic acid)	339.0	75.0* 131.1	0.3	12.0 12.0	20.250
Cis-9,12,15-octadecatrienoic acid (cis-linolenic acid)	335.0	75.1* 131.1 93.1	0.3	21.0 15.0 18.0	20.270
Octadecanoic acid (stearic acid)	341.0	73.0* 325.0	0.3	12.0 12.0	20.440
Cis-11,12-methyleneoctadecanoic acid (phytomonic acid)	353.0	75.1* 131.1	0.3	21.0 12.0	21.190
Nonadecanoic acid (nonadecylic acid)	355.0	75.1* 131.1	0.3	12.0 12.0	21.315
Cis-icosa-5,8,11,14-tetraenoic acid (cis-arachidonic acid)	117.0 91.0 73.0	117.0* 91.0 73.0	0.3	0.0 0.0 0.0	21.490
Eicosanoic acid (arachidic acid)	369.0	75.1* 131.1	0.3	12.0 12.0	22.120
Cis-docosa-4,7,10,13,16,19-hexaenoic acid (cis-docosahexaenoic acid)	117.0 91.0 73.0	117.0* 91.0 73.0	0.3	0.0 0.0 0.0	23.125

*ions selected for quantitative analysis.

#### 3.9.4 Data analysis and optimization of mass spectrometer parameters

The change in the fatty acids’ content in the samples was identified by comparing the ratios of the signal area of the individual analyte to the area of the internal standard (palmitic-d31 acid). Quantitative changes in each of the analyzed fatty acids were compared with control samples (native planktonic cells and native biofilm cells). On this basis, values closer to the data obtained for planktonic and biofilm forms were categorized qualitatively as “similarity with planktonic cells” and “similarity with biofilm cells,” respectively.

In order to optimize mass spectrometry parameters of the method, the analytical standards of fatty acids and IS at a concentration of 100 μg/mL were separately injected and analyzed at the chromatographic conditions described in the “Instrumentation” section in the scan mode in the range of 40–500 m/z. A chromatographic signal, characterized by the chosen example spectra shown in Figure 7 (myristic acid and palmitic acid), were observed at the retention time of 16.715 min and 18.660 min, respectively. The ion with 285 m/z was selected as the precursor ion for myristic acid, while ion with 313 m/z was selected as the precursor ion for palmitic acid. The mentioned precursor ions were then fragmented by collision gas in a collision cell, and the products were analyzed in the scan mode of Q3. The most optimal collision energies (CE) were selected using the MRM method optimization software. The same procedure was applied to other compounds with precursor and product ions identified as those presented in Table 1.

Only for cis-arachidonic acid and cis-docosahexaenoic acid, due to the lack of a precursor ion in the mass spectrometry scan spectra (as can be seen in Figure 7) and a different mechanism of fragmentation, we decided to use characteristic MRM transitions for both these acids, i.e. 117.0 > 117.0, 91.0 > 91.0 and 73.0 > 73.0. Under the chromatographic conditions, the m/z transitions mentioned in Table 1 were selected for optimal monitoring of fatty acids and IS.

### 3.10 Statistics

Statistical analyses were performed using the R program (R-3.4.4 for Windows, CRAN, Vienna, Austria). Normality of data distribution was tested by the Shapiro-Wilk test, while the Kruskal–Wallis test with Holm correction was applied for multiple comparison to check differences between groups. For all the tests, a significance level of α = 0.05 was used.

## 4 Conclusion

As a part of the current research, we demonstrated the modulatory effect of sub-MIC values of antibiotics on the biofilm development of *H. pylori*, with a lack of such phenomenon for MIC values. The biofilm-modulating impact of sub-MICs of MTZ and LEV consisted of an induction of adaptive changes in the membrane fatty acid profile, as well as stimulation of both autoaggregation and the amount of extracellular matrix. When bacteria were exposed to sub-MIC of CLR, the opposite effect was achieved. On this basis, it seems that CLR constitute an antibiotic with a promising biofilm-preventing activity against *H. pylori*. Additionally, we believe the current data adds another layer to our understanding of the importance of maintaining the appropriate concentration of antibiotics during treatment of *H. pylori* as a crucial factor allowing for the achievement of a therapeutic effect. Undoubtedly, studies in animal models or humans are needed to confirm the above observations.

## Data Availability

The original contributions presented in the study are included in the article/Supplementary Material, further inquiries can be directed to the corresponding author.

## Supplementary material

SUPPLEMENTARY VIDEO S1 A 1-h autoaggregation of *H. pylori* 2CML without antibiotic stress (positive control). The autoaggregation experiments took place in 12-well microtiter plates filled with BHI + 5% FCS at 37°C and microaerophilic conditions. The speed of autoaggregation is interpreted as the rate of bacterial coverage of the observation field. For better visualization of the cells, the bacteria were treated with 1 μL/mL of the non-toxic fluorescent dye from the CellTrace CFSE Cell Proliferation Kit.

SUPPLEMENTARY VIDEO S2 The effect of ¼ × MIC of clarithromycin [CLR] on a 1-h autoaggregation of *H. pylori* 2CML. The autoaggregation experiments took place in 12-well microtiter plates filled with BHI + 5% FCS at 37°C and microaerophilic conditions. The speed of autoaggregation isinterpreted as the rate of bacterial coverage of the observation field. For better visualization of the cells, the bacteria were treated with 1 μL/mL of the non-toxic fluorescent dye from the CellTrace CFSE Cell Proliferation Kit.

SUPPLEMENTARY VIDEO S3 The effect of ¼ × MIC of metronidazole [MTZ] on a 1-h autoaggregation of *H. pylori* 2CML. The autoaggregation experiments took place in 12-well microtiter plates filled with BHI + 5% FCS at 37°C and microaerophilic conditions. The speed of autoaggregation isinterpreted as the rate of bacterial coverage of the observation field. For better visualization of the cells, the bacteria were treated with 1 μL/mL of the non-toxic fluorescent dye from the CellTrace CFSE Cell Proliferation Kit.

SUPPLEMENTARY VIDEO S4 The effect of ¼ × MIC of levofloxacin [LEV] on a 1-h autoaggregation of *H. pylori* 2CML. The autoaggregation experiments took place in 12-well microtiter plates filled with BHI + 5% FCS at 37°C and microaerophilic conditions. The speed of autoaggregation isinterpreted as the rate of bacterial coverage of the observation field. For better visualization of the cells, the bacteria were treated with 1 μL/mL of the non-toxic fluorescent dye from the CellTrace CFSE Cell Proliferation Kit.
