# Supplementary material for: Subinhibitory concentrations of antibiotics affect development and parameters of Helicobacter pylori biofilm
Source: Front Pharmacol. 2024 Oct 14;15:1477317. doi: 10.3389/fphar.2024.1477317 (PMC11513322; doi:10.3389/fphar.2024.1477317)
Supplement: Supplementary file 2 [file Presentation1.pdf]

Supplementary Materials

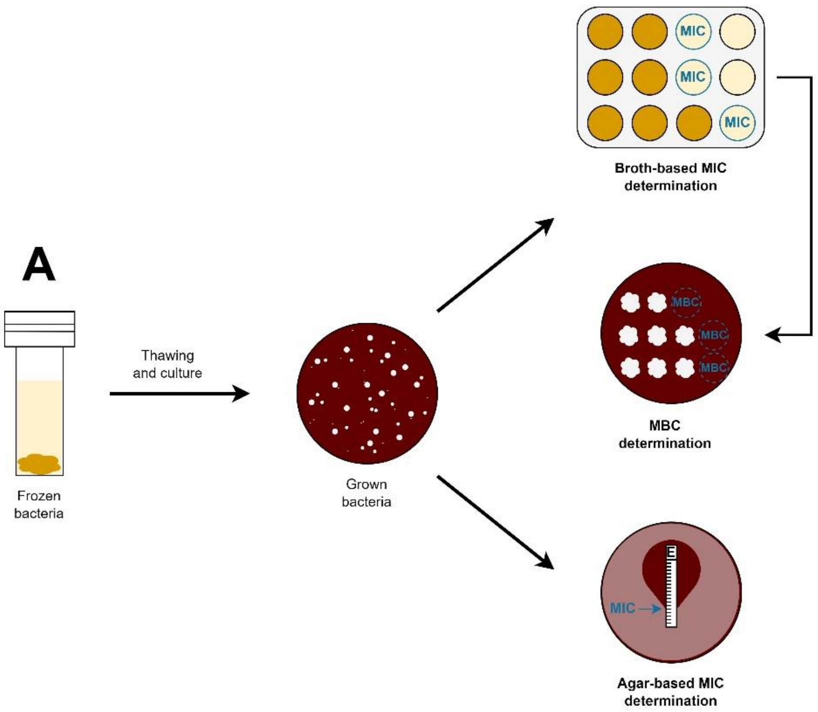

B

Bacterial strains	Broth-based MIC determination [µg/mL]		
	CLR	MTZ	LEV
H. pylori 1CML	8	64	4
H. pylori 2CML	16	64	16
H. pylori 3CML	2	128	16

C

Bacterial strains	Broth-based MBC determination [µg/mL]		
	CLR	MTZ	LEV
H. pylori 1CML	16	256	32
H. pylori 2CML	32	128	16
H. pylori 3CML	4	256	32

D

Bacterial strains	Agar-based MIC determination [µg/mL]		
	CLR	MTZ	LEV
H. pylori 1CML	24	256	32
H. pylori 2CML	256	256	32
H. pylori 3CML	24	256	32

Figure S1. **Determination of minimal inhibitory concentration (MIC) and minimal bactericidal concentration (MBC) vales of antibiotics against *H. pylori*.** Cartoon images **(A)** showing the procedure determining the antibiotic sensitivity of *H. pylori* using two different techniques: broth-based and agar-based. Table **B** and **C** presents MIC and MBC values of the tested *H. pylori* strains obtained by the broth-based method. Table **D** shows MIC values of the tested *H. pylori* strains obtained by the agar-based method. Obtaining MBCs using the agar-based method is not possible. Abbreviations: CLR, clarithromycin; MTZ, metronidazole; LEV, levofloxacin.

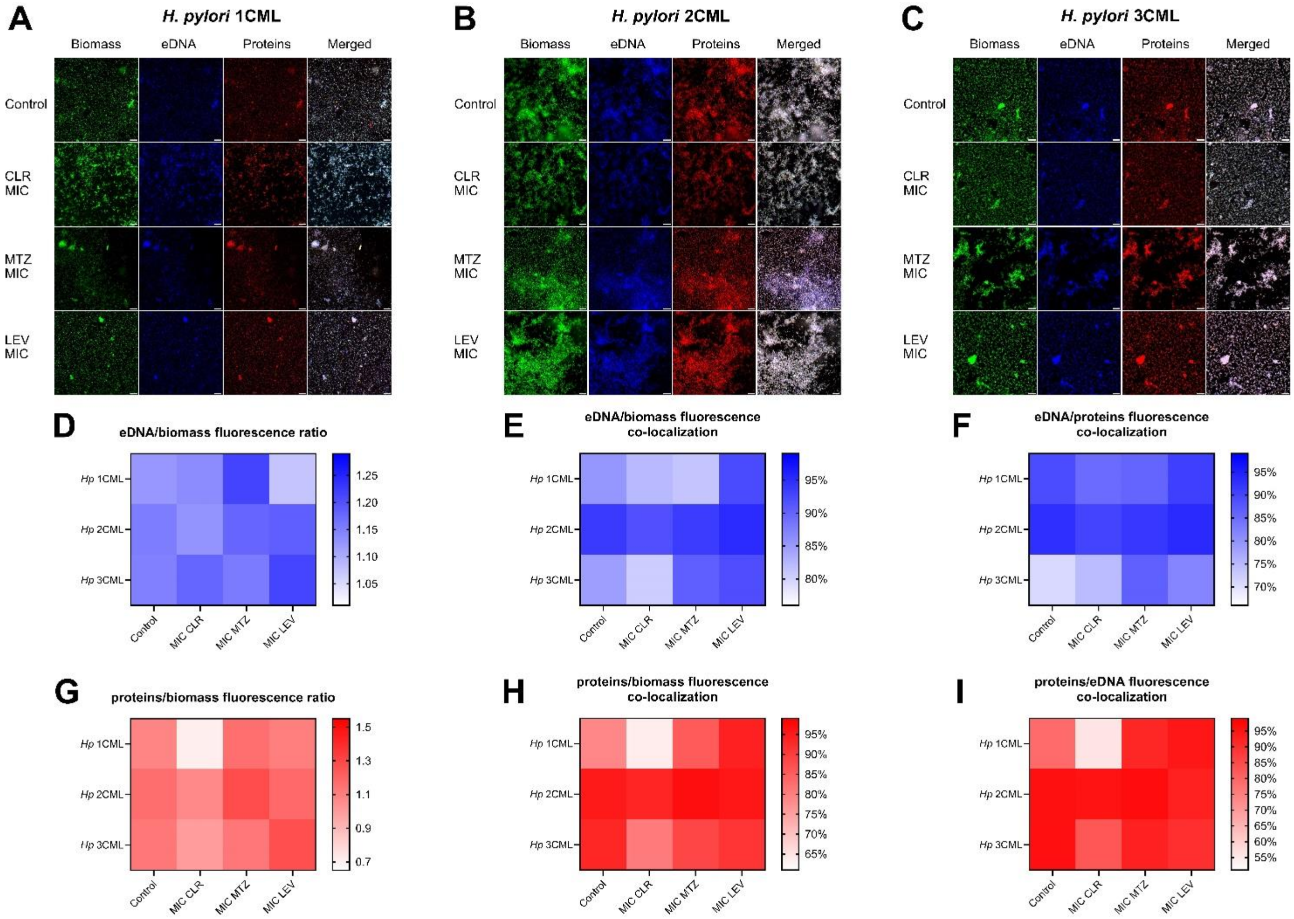

Figure S2. **The effect of antibiotics on the biochemical properties of the developing biofilm of *H. pylori*.** Schematic panels illustrating representative images of 3-day-old biofilms of *H. pylori* 1CML **(A)**, 2CML **(B)**, and 3CML **(C)** developed under constant exposure to MIC of antibiotics (clarithromycin [CLR], metronidazole [MTZ] and levofloxacin [LEV]). The development of biofilms took place in 12-well microtiter plates filled with BHI + 5% FCS for 3 days at 37 ^{o}C, microaerophilic conditions and shaking at 50 rpm. Cell biomass, eDNA and proteins were fluorescently stained with SYTO9, DAPI and SYPRO RUBY, respectively. Scale bars = 40 µm. Using the Bioflux Montage software, the fluorescence intensity of biofilm components **(D,G)** and the degree of their co-localization **(E,F,H,I)** was calculated and the obtained data were presented as heat maps. Data for eDNA are shown in points **D-F**, while data for proteins are illustrated in points **G-I**. In all cases, values obtained are from three biological replicates with five technical replicates (n = 15).

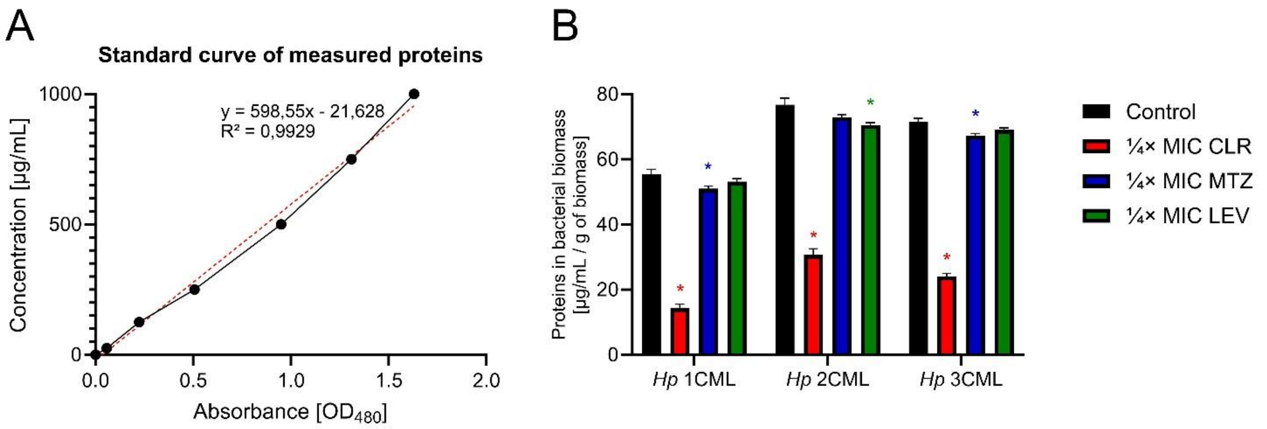

Figure S3. **The effect of antibiotics on the proteins amount in bacterial biomass of *H. pylori*.** The amount of proteins was determined using a Rapid Gold Pierce BCA protein assay and a spectrophotometric quantification at a wavelength of 480 nm (OD_{480}). Standard curve of the amount of analyzed proteins **(A)**, which was determined experimentally on the basis of albumin (0 – 1 mg/mL) included in the kit. Graph showing the amount of proteins in bacterial biomass **(B)** after exposure to ¼× MIC of antibiotics (clarithromycin [CLR], metronidazole [MTZ] and levofloxacin [LEV]). Bacteria were cultured in 12-well microtiter plates filled with BHI + 5% FCS for 3 days at 37 °C, microaerophilic conditions and shaking at 100 rpm. The obtained results were normalized to the weight of the dry bacterial biomass. The tests were performed in three biological replications with three technical repetitions (n = 9/strain). Statistical analysis was performed using the Kruskal-Wallis test with Holm correction. $p < 0.05$ was considered statistically significant.

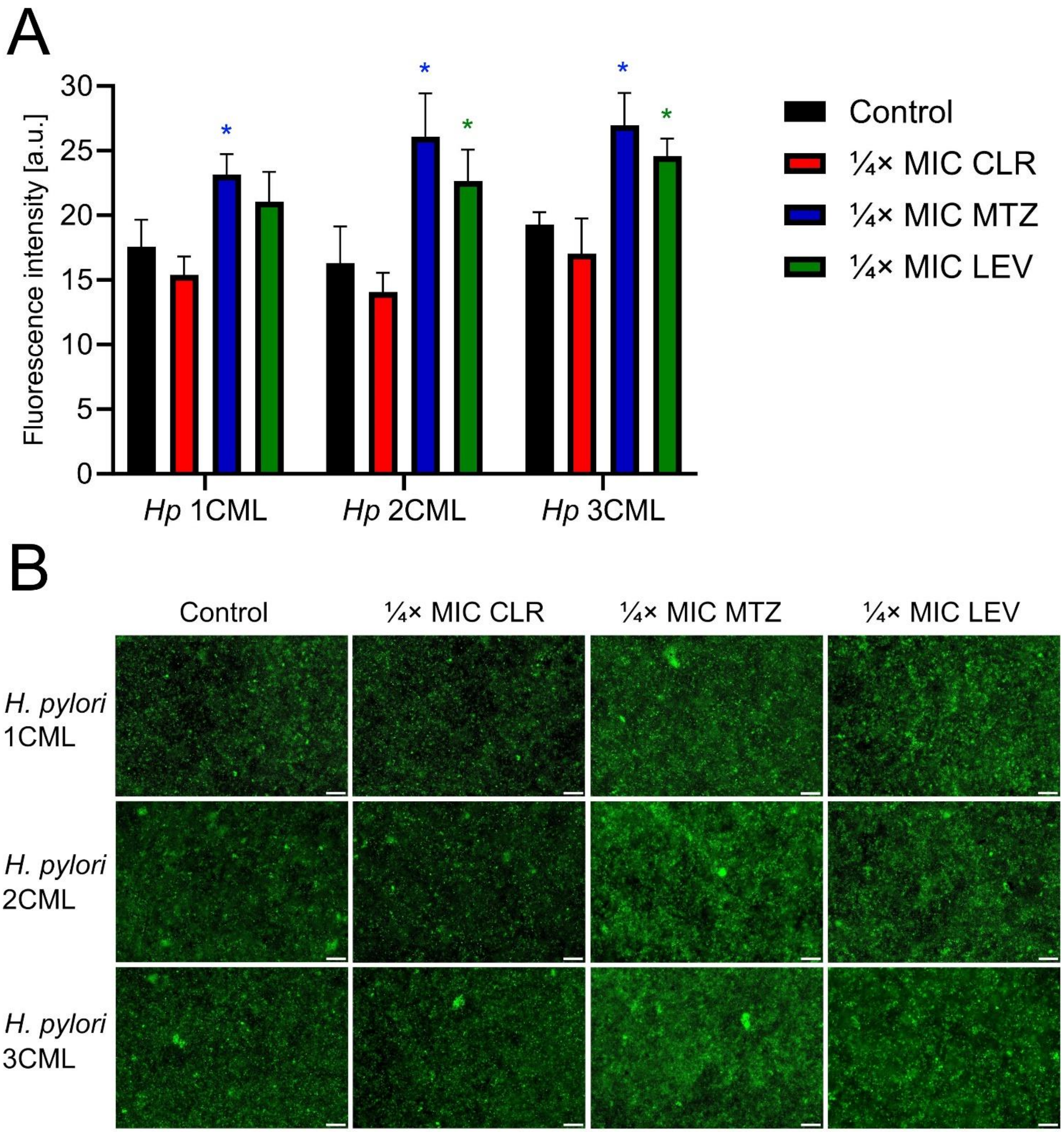

Figure S4. **The effect of antibiotics on the oxidative stress in bacterial biomass of *H. pylori*.** The oxidative stress level in bacterial cells was determined using a H2DCFDA staining and fluorescence estimation during microscopic observations. Graph **(A)** and panel of representative photos **(B)** showing the level of oxidative stress in bacterial cells after exposure to ¼× MIC of antibiotics (clarithromycin [CLR], metronidazole [MTZ] and levofloxacin [LEV]). Bacteria were cultured in 12-well microtiter plates filled with BHI + 5% FCS for 3 days at 37 ^{o}C, microaerophilic conditions and shaking at 100 rpm. The tests were performed in three biological replications with three technical repetitions constituting different observation fields of the examined glass slide (n = 9/strain). Statistical analysis was performed using the Kruskal-Wallis test with Holm correction. $p < 0.05$ was considered statistically significant.

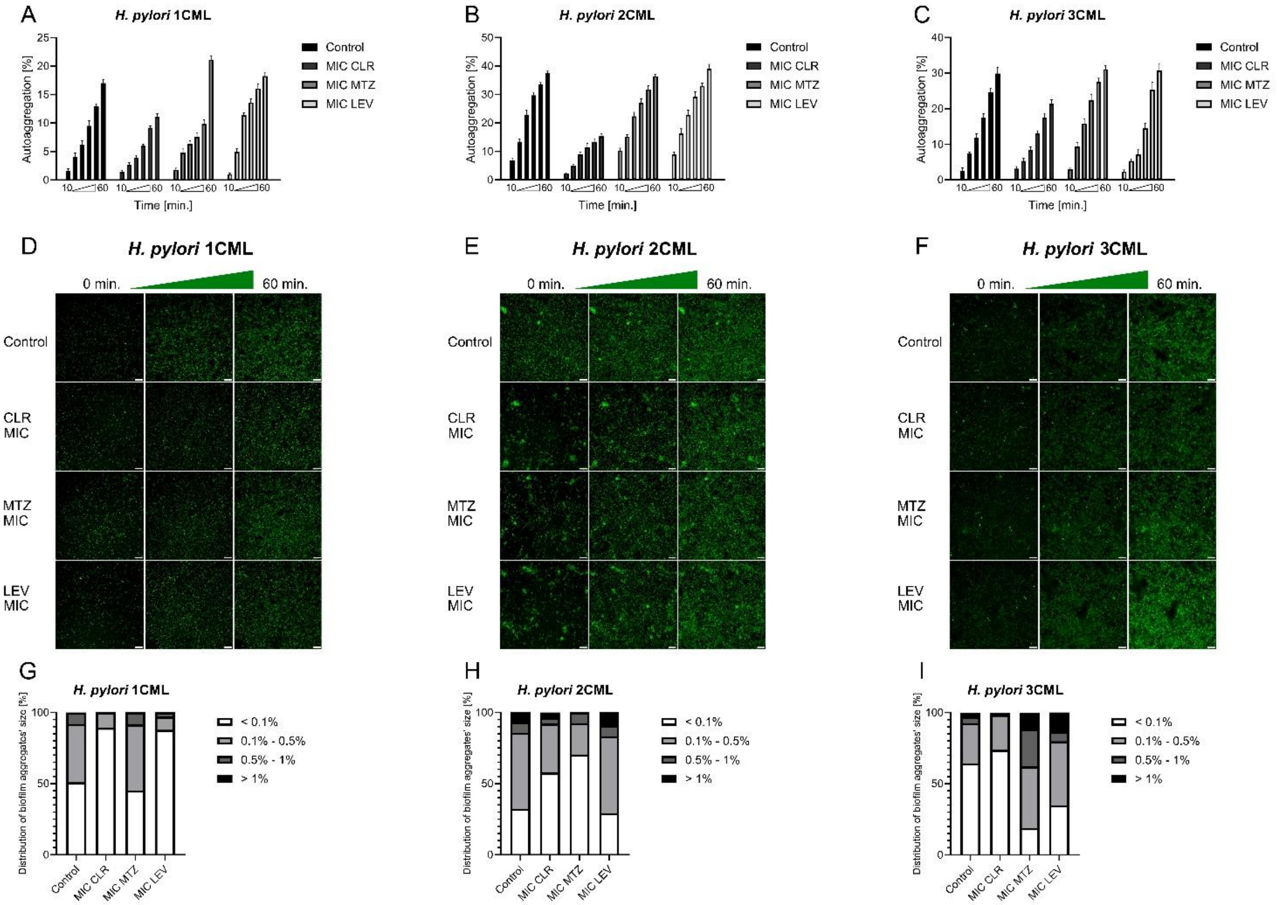

Figure S5. **The effect of antibiotics on the autoaggregation of *H. pylori*.** Graphs and panels of representative photos showing the speed of autoaggregation of *H. pylori* 1CML **(A,D)**, 2CML **(B,E)** and 3CML **(C,F)** during 1-h exposure to MIC of antibiotics (clarithromycin [CLR], metronidazole [MTZ] and levofloxacin [LEV]). The autoaggregation experiments took place in 12-well microtiter plates filled with BHI + 5% FCS at 37 ^{o}C and microaerophilic conditions. The speed of autoaggregation was interpreted as the rate of bacterial coverage of the observation field and was calculated using the ImageJ software. The speed of autoaggregation was each time normalized in relation to the initial amount of autoaggregation (0 min.), hence the starting point was not included in graphs **A-C** and was considered as 0%. For better visualization of the cells, the bacteria were treated with 1 µL/mL of the non-toxic fluorescent dye from the CellTrace CFSE Cell Proliferation Kit. Scale bars for photographic panels **(D-F)** are equal to 40 µm. Using the ImageJ software, additional information was obtained about the size distribution of aggregates, which was presented in **G-I** charts. In all cases, values obtained are from three biological replicates with three technical replicates (n = 9); the results are the mean ± standard deviations. Statistical analysis was performed using the Kruskal-Wallis test with Holm correction. The data was not statistically different.

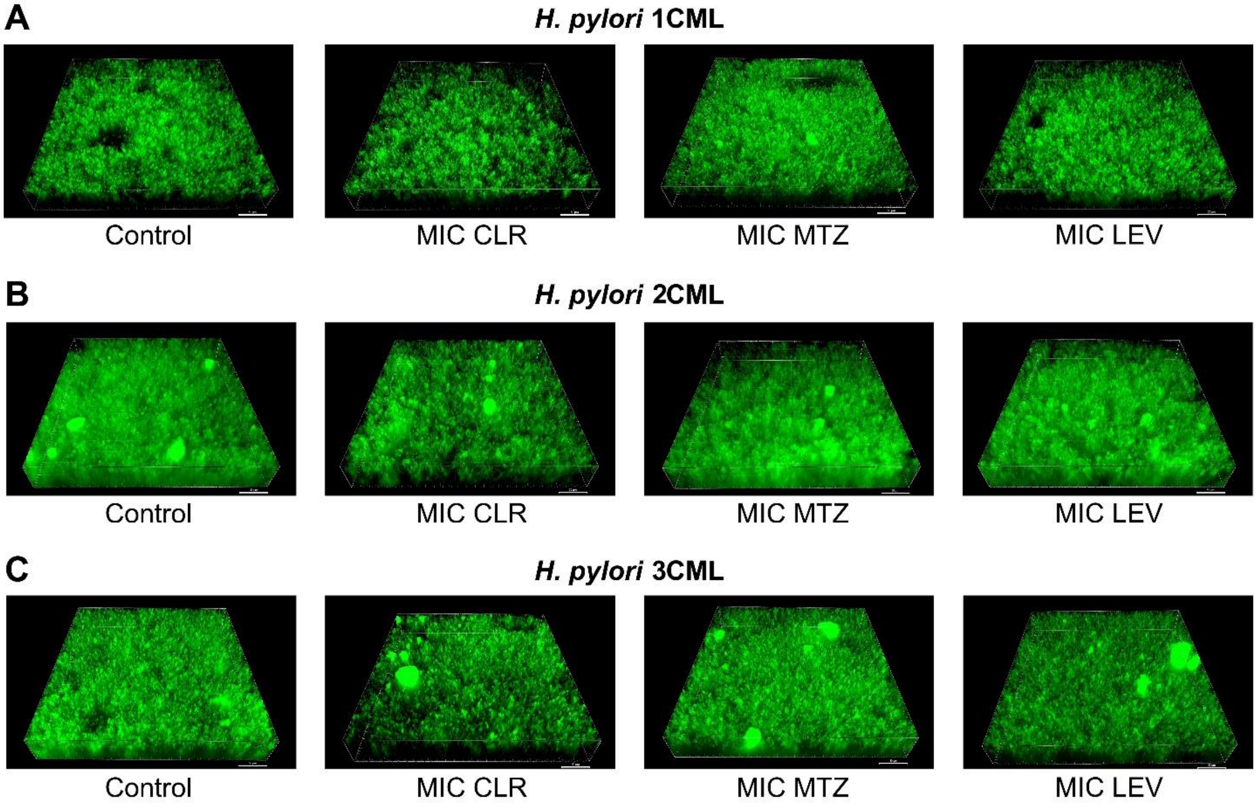

Figure S6. **The effect of antibiotics on the biophysical properties of the developing biofilm of *H. pylori***. Panels of representative photos showing biofilms of *H. pylori* 1CML **(A)**, 2CML **(B)** and 3CML **(C)** created during autoaggregation experiments and 1-h exposure of bacteria to MIC of antibiotics (clarithromycin [CLR], metronidazole [MTZ] and levofloxacin [LEV]). The development of biofilms took place in 12-well microtiter plates filled with BHI + 5% FCS at 37 °C and microaerophilic conditions. For visualization of the bacterial biomass, cells were treated with 1 µL/mL of the non-toxic fluorescent dye from the CellTrace CFSE Cell Proliferation Kit. Using the Bioflux Montage software, a total of 50 Z-stacks were collected in each case from the center of the examined well of the microtiter plate. The photographs obtained in this way were processed using the ImarisViewer. Scale bars = 50 µm.

Table S1. **Differences in the profile of selected fatty acids building cell membranes of *H. pylori* in control samples and samples exposed to ¼× MIC of LEV.**

**Bacterial strains**	**Tested conditions**	**C18:1/C18:0**		**C18:2/C18:0**		**C19c:0/C18:0**	
		**Ratio**	**Fold change**	**Ratio**	**Fold change**	**Ratio**	**Fold change**
H. pylori 1CML	Control	6.68	0.75	4.4	0.2	16.27	**1.3**
	LEV	5.02		0.9		21.16	
H. pylori 2CML	Control	1.06	6.07	0.19	6	4.8	**5.2**
	LEV	6.43		1.14		24.97	
H. pylori 3CML	Control	7.12	3.18	1.99	2.52	80.96	**2.13**
	LEV	22.63		5.01		172.38	

Bacteria were cultured in 12-well microtiter plates filled with BHI + 5% FCS for 3 days at 37 °C, microaerophilic conditions and shaking at 100 rpm. Bolded values indicate an increase in the ratio of a given fatty acid after antibiotic treatment (¼× MIC of LEV) in all three *H. pylori* strains. Legend: LEV, levofloxacin; C18:0, octadecanoic acid (stearic acid); C18:1, cis-9-octadecenoic acid (cis-oleic acid); C18:2, cis-9,12-octadecadienoic acid (cis-linoleic acid); C19c:0, cis-11,12-methyleneoctadecanoic acid (phytomonic acid).
